# Bridging the gap between statistical significance and clinical relevance: A systematic review of minimum clinically important difference (MCID) thresholds of scales reported in movement disorders research

**DOI:** 10.1016/j.heliyon.2024.e26479

**Published:** 2024-02-20

**Authors:** Biswamohan Mishra, Pachipala Sudheer, Roopa Rajan, Ayush Agarwal, M V Padma Srivastava, Nilima Nilima, Venugopalan Y. Vishnu

**Affiliations:** a Department of Neurology, All India Institute of Medical Sciences, New Delhi, India; b Department of Biostatistics, All India Institute of Medical Sciences, New Delhi, India

**Keywords:** Parkinsonism, Dystonia, Tremor, Tardive dyskinesia, Ataxia, Movement disorders, Minimal clinical important difference, Minimal clinically important change, Clinical relevance, Patient-reported outcome measures (PROMs), MCID

## Abstract

**Background:**

Minimum clinically important difference **(**MCID) is the smallest change in an outcome measure that is considered clinically meaningful. Using validated MCID thresholds for outcomes powers trials adequately to detect meaningful treatment effects, aids in their interpretation and guides development of new outcome measures.

**Objectives:**

To provide a comprehensive summary of MCID thresholds of various symptom severity scales reported in movement disorder.

**Methods:**

We conducted systematic review of the literature and included studies of one or more movement disorders, and reporting MCID scales.

**Results:**

2763 reports were screened. Final review included 32 studies. Risk of bias (RoB) assessment showed most studies were of good quality. Most commonly evaluated scale was Unified Parkinson's Disease Rating Scale (UPDRS) (11 out of 32). Four studies assessing MDS-UPDRS had assessed its different sub-parts, reporting a change of 2.64,3.05,3.25 and 0.9 points to detect clinically meaningful improvement and 2.45,2.51,4.63 and 0.8 points to detect clinically meaningful worsening, for the Part I, II, III and IV, respectively. For Parts II + III, I + II + III and I + II + III + IV, MCID thresholds reported for clinically meaningful improvement were 5.73, 4.9, 6.7 and 7.1 points respectively; while those for clinically meaningful worsening were 4.7, 4.2, 5.2 and 6.3 points, respectively. MCID thresholds reported for other scales included Abnormal Involuntary Movement Scale (AIMS), Toronto Western Spasmodic Torticollis Rating Scale (TWSRS), and Burke-Fahn-Marsden Dystonia Scale (BFMD).

**Conclusion:**

This review summarizes all the MCID thresholds currently reported in Movement disorders research and provides a comprehensive resource for future trials, highlighting the need for standardized and validated MCID scales in movement disorder research.

Movement disorders, such as Parkinson's disease, essential tremor, dystonia, and Huntington's disease, can significantly impair a person's quality of life, physical function, and overall well-being. Measuring the effectiveness of interventions, including medication, surgical, and rehabilitation approaches, is crucial in these conditions. Clinical trials and studies often use rating scales to assess the severity of symptoms and to evaluate the impact of interventions [1]. However, simply measuring changes in scores may not fully reflect the meaningfulness of the treatment effect on patients' lives. Therefore, determining the Minimal Clinically Important Difference (MCID) is crucial to establish the smallest amount of change in symptom severity that is meaningful to patients [2], first described by Jaeschke and colleagues in 1989 [3]. The MCID is a valuable psychometric measure that helps researchers design and interpret therapeutic trials, clinicians to evaluate treatment efficacy, and patients to understand and make informed decisions about their care [4].

The MCID (minimum clinically important difference) represents the smallest change in a measurement that is clinically meaningful to patients [5]. The two most commonly used methods for estimation of MCID are the anchor-based method, and the distribution-based method. The former examines the relationship between the change in score at hand and another external measure of clinical change: the anchor, which may be a clinical outcome or a Patient Reported Outcome (PRO). The latter relies on the statistical properties of the distribution of outcome scores, especially, how widely the scores are distributed between patients. The Delphi method has also been proposed, which involves the presentation of a questionnaire to a panel of experts, the opinions of whom are averaged, and the process is repeated, until a consensus is achieved [6]. Different methods give different estimates of MCID, and the method used should be individually determined in the context of each unique clinical study.

In movement disorder trials, understanding the MCID is important for several reasons including determining treatment efficacy, interpreting clinical trial results, planning future trials and guiding clinical practice. For example, testing for the efficacy of a new medication using the MDS (Movement Disorder Society) - UPDRS scale, wherein the MCID for Part III has been supposedly estimated at 3–5 points. If the treatment group shows a statistically significant reduction in UPDRS motor score compared to the placebo group, but the difference is less than 3–5 points, it may not be clinically meaningful and further investigation may be necessary. Thus, incorporating MCID into movement disorders trials can improve the quality of research and enhance the care of patients with movement disorders.

This review aims to familiarize clinicians with the definition and methods for estimation of MCID, different patient-reported and clinician-assessed symptom severity scales in movement disorders, and their use in the calculation of MCID for movement disorder-related scales.

## 1 Methods

### 1.1 Literature search

A literature search was performed using the MEDLINE, Embase, and Cochrane Library databases to find studies reporting MCID thresholds in various movement disorders. The following search criteria was used: “MCID” OR “MID” OR “minimal clinically important difference” OR “minimal important difference” OR “minimal clinically important change” OR “clinically important change” OR “minimal clinical important difference” OR “clinical important difference” OR “meaningful change” AND “parkinsonism” OR “dystonia” OR “essential tremor” OR “tardive dyskinesia” OR “ataxia” OR “Spasmodic dysphonia” OR “Chorea” OR “Hemiballismus” OR "Huntington's disease,” OR “Tourette syndrome,” OR “Restless legs syndrome,” OR “Multiple system atrophy,” OR “Progressive supranuclear palsy,” OR “Cortico-basal degeneration.” The reference lists of the assessed articles were also searched for the relevant studies. In case of non-availability of full texts, the corresponding authors were contacted for full texts. This systematic review was performed in accordance with PRISMA (Preferred Reporting Items for Systematic Reviews and Meta-Analyses) guidelines. The protocol for this review is registered with the International Prospective Register of Systematic Reviews (PROSPERO CRD42023408999).

### 1.2 Study selection

We included both randomized controlled trials and observational studies that report MCID thresholds for commonly used symptom severity scales in movement disorders.

Studies meeting all of the following criteria were included:

• Randomized controlled trials (RCTs), Non-randomized controlled trials (NRCTs), Cohort, Case-control studies, Cross-sectional studies or Other observational studies (e.g. case series) that report primary data and investigate or report on MCID in movement disorders
• Studies published in English language

Studies meeting any of the following criteria were excluded:

• Case Reports
• Studies that involve animal models or in vitro studies
• Studies that only report secondary data (e.g. review articles, commentaries)

### 1.3 Data extraction

Two reviewers (BM and AA) independently screened the titles and abstracts of identified studies to determine eligibility for inclusion. Full-text articles of potentially relevant studies were obtained and reviewed to determine final eligibility. Data extraction was conducted independently by these two reviewers using a standardized data extraction form. The following data were extracted from each study: study design, population characteristics, intervention/exposure details, primary and secondary outcomes, MCID thresholds and methods of calculation, and any other relevant data. Any discrepancies between the two reviewers were resolved through discussion and consensus with a third reviewer (RR). Data was recorded in Microsoft excel version 16.72 for further analysis.

### 1.4 Main outcomes

• To identify and summarize the reported MCID thresholds of various symptom severity/assessment scales reported in movement disorders trials.
• To review disease-specific rating scales, functional measures, and patient-reported outcome measures, which have been used to assess the effectiveness of interventions in movement disorders.

### 1.5 Additional outcomes

• To examine whether the MCID thresholds vary across different movement disorder conditions, such as Parkinson's disease, dystonia, essential tremor, ataxia, Huntington's disease, Tourette syndrome, Restless legs syndrome, Chorea, Athetosis, Tardive dyskinesia, Multiple system atrophy, Progressive supranuclear palsy, Corticobasal degeneration, Spasmodic dysphonia, and Hemiballismus.
• To investigate whether the MCID thresholds vary according to patient characteristics, such as age, sex, disease duration, and severity.
• To assess the quality of reporting of MCID thresholds in the trials included in the review.
• To identify any gaps in the current literature on MCID thresholds in trials of movement disorders.

### 1.4 Risk of bias assessment

The risk of bias of the included studies was assessed using the Cochrane Risk of Bias tool for randomized controlled trials (RCTs) [7] and the Joanna Briggs Institute Critical Appraisal Checklist for observational studies [8]. For those studies which analyzed the data from previously conducted RCTs, the RoB using the Cochrane Risk of Bias tool for randomized controlled trials, was assessed for on the original studies. Two reviewers (BM and AA) independently assessed the risk of bias of each included study, and any discrepancies was resolved through discussion and consensus.

### 1.5 Data synthesis

The data synthesis for this systematic review was conducted in a narrative format, as the heterogeneity of the included studies precluded a quantitative meta-analysis. The data extracted from the included studies was summarized and presented in tables and figures to facilitate comparisons and identification of patterns across studies. The tables include information on study design, sample size, population characteristics, intervention/exposure details, MCID thresholds and methods of calculation, and any other relevant data.

## 2 Results

### 2.1 Study selection

**Fig. 1:**
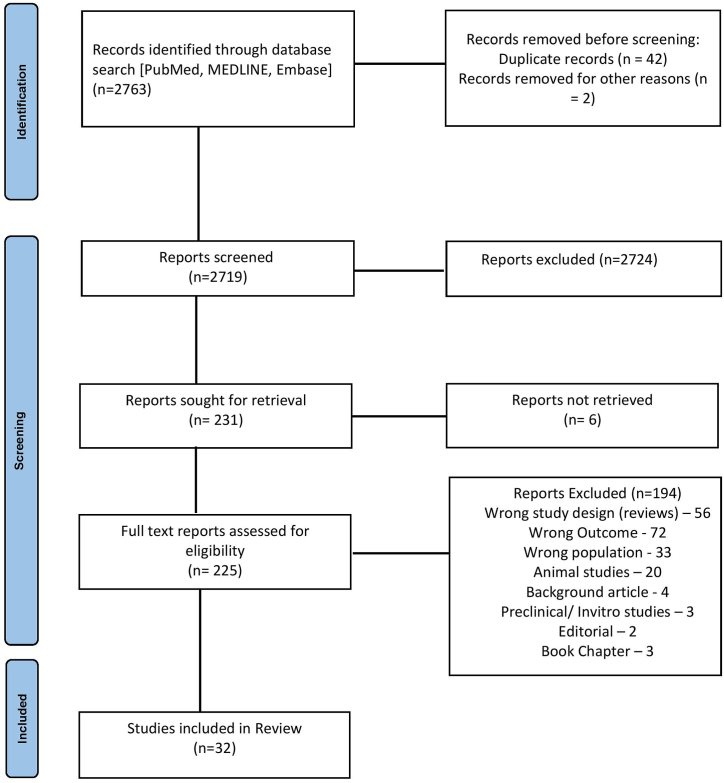
PRISMA (Preferred Reporting Items for Systematic Reviews and Meta-analyses) flowchart showing the identification, screening, and inclusion of trials for this systematic review and meta-analysis.

Literature search using the above-mentioned method revealed 2763 records. After screening and excluding the records not relevant to our study, a total of 32 studies were included in the review. (Fig. 1). There were 10 RCTs and 22 non-RCTs included in these 32 studies.

### 2.2 Risk of bias assessment (RoB)

**Fig. 2:**
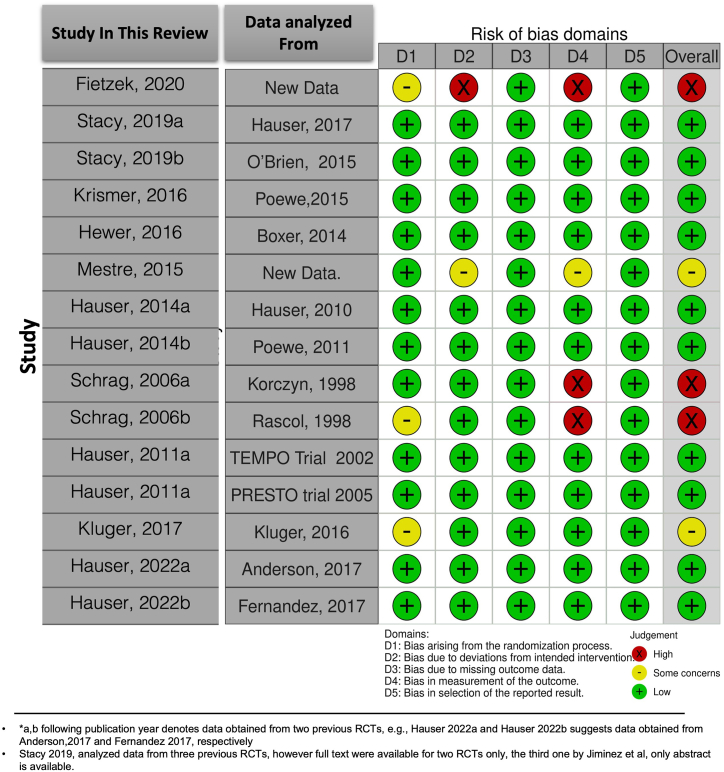
Shows the Traffic light plot representation of risk of bias assessment of the Randomized Controlled trials included in this systematic review using the Revised Cochrane risk-of-bias tool for randomized trials (RoB2).

**Fig. 3:**
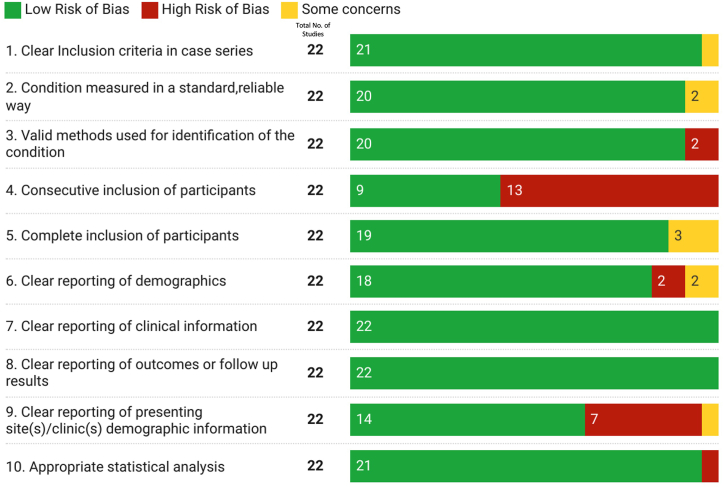
Shows Traffic light plot representation of risk of bias assessment of the Randomized Controlled trials included in this systematic review using the Revised Cochrane risk-of-bias tool for randomized trials (RoB2).

Among the RCTs, most of the studies had a low risk of bias (six out of 10), two had some concerns and two studies had high RoB (Domain 6 -Overall RoB, Fig. 2 and Table 1). A major source of bias occurred in Domain 4 (Risk of bias in measurement of the outcome). Among the non-RCTs included in the review, majority of studies were assessed to be of low risk of bias in all the domains examined (green bars in Fig. 3), except domain 4 i.e., ‘Did the case series have consecutive inclusion of participants?’ (high RoB – 13 out of 22 studies) and domain 9 i.e., ‘Was there clear reporting of the presenting site(s)/clinic(s) demographic information?’ (high RoB – 7 out of 22 studies) (Fig. 3, Table 2). Overall, the quality of studies included in this study were of acceptable quality.

**Table 1:** Showing risk of bias assessment of the Randomized Controlled trials included in this systematic review using the Revised Cochrane risk-of-bias tool for randomized trials (RoB2).

Sl. No.	Signalling Questions		Domain 1. Randomization process	Domain 2. Deviations from intended interventions	Domain 3. Missing outcome data	Domain 4. Measurement of the outcome	Domain 5. Selection of the reported result	Domain 6. Overall Bias
	**Author, Year of included RCTs**	**Data for analysis sourced from**	1.0 Assessor's Judgement	2.0 Assessor's Judgement	3.0 Assessor's Judgement	4.0 Assessor's Judgement	5.0 Assessor's Judgement	Assessor's overall Judgement
1	**Fietzek et al, 2020** [9]		SOME CONCERNS	HIGH	LOW	HIGH	LOW	HIGH
2	**Stacy et al, 2019** [10]	**Hauser et al., 2017** [86]	LOW	LOW	LOW	LOW	LOW	LOW
		**O'Brien et al., 2015** [87]	LOW	LOW	LOW	LOW	LOW	LOW
3	**Krismer et al, 2016** [11]	**Poewe et al., 2015** [88]	LOW	LOW	LOW	LOW	LOW	LOW
4	**Hewer et al, 2016** [12]	**Boxer et al., 2014** [89]	LOW	LOW	LOW	LOW	LOW	LOW
5	**Mestre et al, 2015** [13]		LOW	SOME CONCERNS	LOW	SOME CONCERNS	LOW	SOME CONCERNS
6	**Hauser et al, 2014** [14]	**Hauser et al., 2010** [90]	LOW	LOW	LOW	LOW	LOW	LOW
		**Poewe et al., 2011** [91]	LOW	LOW	LOW	LOW	LOW	LOW
7	**Schrag et al, 2006** [15]	**Korczyn et al., 1998** [92]	LOW	LOW	LOW	HIGH	LOW	HIGH
		**Rascol et al., 1998** [93]	SOME CONCERNS	LOW	LOW	HIGH	LOW	HIGH
8	**Hauser et al, 2011** [16]	**TEMPO Trial Parkinson Study Group 2002** [94]	LOW	LOW	LOW	LOW	LOW	LOW
		**PRESTO trial Parkinson Study Group 2002** [95]	LOW	LOW	LOW	LOW	LOW	LOW
9	**Kluger et al., 2017** [17]	**Kluger et al., 2016** [96]	SOME CONCERNS	LOW	LOW	LOW	LOW	SOME CONCERNS
10	**Hauser et al., 2022** [18]	**Anderson et al., 2017** [97]	LOW	LOW	LOW	LOW	LOW	LOW
		**Fernandez et al., 2017** [98]	LOW	LOW	LOW	LOW	LOW	LOW

Overall risk of bias judgement.

Low risk: The study is judged to be at low risk of bias for all domains for this result.

Some concerns: The study is judged to raise some concerns in at least one domain for this result, but not to be at high risk of bias for any domain.

High risk of bias: The study is judged to be at high risk of bias in at least one domain for this result.

**Table 2:** Showing risk of bias assessment of the Non-Randomized studies included in this systematic review using Joanna Briggs Institute's (JBI) Critical Appraisal Checklist.

Sl. No.	Study name, Year	1. Clear Inclusion criteria in case series	2. Condition measured in a standard, reliable way	3. Valid methods used for identification of the condition	4. Consecutive inclusion of participants	5. Complete inclusion of participants	6. Clear reporting of demographics	7. Clear reporting of clinical information	8. Clear reporting of outcomes or follow up results	9. Clear reporting of presenting site(s)/clinic(s) demographic information	10. Appropriate statistical analysis
1	**Pinter et al, 2019** [19]	Yes	Yes	Yes	Yes	Yes	Yes	Yes	Yes	Yes	Yes
2	**Espay et al, 2018** [20]	Yes	Yes	Yes	No	Unclear	Yes	Yes	Yes	No	Yes
3	**Dashtipour et al, 2019** [21]	Yes	Yes	Yes	No	Yes	Yes	Yes	Yes	Yes	Yes
4	**Pinter et al, 2020** [22]	Yes	Yes	Yes	Yes	Yes	Yes	Yes	Yes	Yes	Yes
5	**Barkey et al, 2021** [23]	Yes	Yes	Yes	No	Unclear	Unclear	Yes	Yes	No	Yes
6	**Godi et al, 2020** [24]	Yes	Yes	Yes	Yes	Yes	Yes	Yes	Yes	Yes	Yes
7	**Henderson et al, 2019** [25]	Yes	Yes	Yes	Not Applicable	Yes	Yes	Yes	Yes	Yes	Yes
8	**Makkos et al, 2019** [1]	Yes	Yes	Yes	No	Yes	Yes	Yes	Yes	No	Yes
9	**Sanchez-Ferro et al, 2018** [26]	Yes	Unclear	No	No	Unclear	No	Yes	Yes	No	No
10	**Makkos et al, 2018** [27]	Yes	Yes	Yes	No	Yes	Yes	Yes	Yes	No	Yes
11	**Horvath et al, 2017** [28]	Yes	Yes	Yes	No	Yes	Yes	Yes	Yes	No	Yes
12	**Horvath et al, 2017** [29]	Yes	Yes	Yes	No	Yes	Yes	Yes	Yes	Yes	Yes
13	**Horvath et al, 2015** [30]	Yes	Yes	Yes	Yes	Yes	Yes	Yes	Yes	Yes	Yes
14	**Horvath et al, 2015** [31]	Yes	Yes	Yes	Yes	Yes	Yes	Yes	Yes	Yes	Yes
15	**Luo et al, 2009** [32]	Yes	Yes	Yes	Yes	Yes	Unclear	Yes	Yes	Yes	Yes
16	**Martinez-Martin et al, 2006** [33]	Yes	Yes	Yes	No	Yes	Yes	Yes	Yes	Unclear	Yes
17	**Fitzpatrick et al, 2004** [34]	Yes	Yes	Yes	Yes	Yes	Yes	Yes	Yes	Yes	Yes
18	**Shulman et al, 2010** [35]	Yes	Yes	Yes	No	Yes	Yes	Yes	Yes	Yes	Yes
19	**Steffen and seney et al, 2008** [36]	Yes	Yes	Yes	No	Yes	No	Yes	Yes	Yes	Yes
20	**Peto et al, 2001** [37]	Unclear	Unclear	No	Not Applicable	Yes	Yes	Yes	Yes	No	Yes
21	**Honig et al, 2009** [38]	Yes	Yes	Yes	No	Yes	Yes	Yes	Yes	Yes	Yes
22	**Esther Cubo et al., 2021** [39]	Yes	Yes	Yes	No	Yes	Yes	Yes	Yes	Yes	Yes

### 2.3 Results on the MCID thresholds reported in the included studies

Majority of studies reported on MCID thresholds of the Unified Parkinson's Disease Rating Scale (UPDRS) scale (eleven out of 32 studies). Three studies each reported on Abnormal involuntary movement scale (AIMS), and Parkinson's disease (PD) “quality of life” questionnaires (the PDQ-39 and PDQ-8). Two studies each reported on Toronto Western Spasmodic Torticollis Rating Scale (TWSTRS), Unified Dyskinesia Rating Scale (UDysRS), and Burke-Fahn-Marsden Dystonia Rating Scale (BFMDRS). Multiple other scales were also evaluated by various other singular studies as described below. A summary of the various MCID thresholds along with patient characteristics, method used to estimate MCID, and the anchors and distribution criteria used are provided in Table 3, Table 4 and Table 5. The results have been divided into two sections, the first one discussing the MCID thresholds of scales related to PD and the subsequent one of scales pertaining to other movement disorders.

**Table 3:** Shows the characteristics of the population, including disease duration and severity of the disease in the studies included (the first half shows studies assessing patients with Parkinson's disease while the second half shows studies assessing various other movement disorders.

Table 3.1 shows studies assessing patients with Parkinson's disease												
S no.	Author, Year	No. of patients	Mean Age	Disease (specific treatment)	Disease Duration in years	Mean MDS UPDRS Score at baseline in points	Disease Severity in % (of the total participants)	Levodopa Usage in years	Mean LED in mg	Off Period in hours	Fluctuations duration in years	Whether assessed in On and/Off period
1	Makkos et al., 2019 [1]	436	–	Parkinson's disease (Medical, DBS)	8.0 ± 6	71.4 ± 27.5 (I + II + III + IV)	Mild (HYS 1 and 2) −61.9% Moderate (HYS 3) – 24.3% Severe (HYS 4 and 5) −13.8%	6.3 ± 5.6	839.9 ± 637.7		4.2 ± 3.9	NI on MDS UPDRS MDS UDyRS assessed in both On and Off period
2	Makkos et al., 2018 [27]	452	–	Parkinson's disease (treatment not mentioned/probably usual care)	7.4 ± 6.3	72.9 ± 25.1 (I + II + III + IV)	Mild (HYS 1 and 2) −46.7% Moderate (HYS 3) – 34.5% Severe (HYS 4 and 5) −19.0%	–	750.4 ± 615		4.8 ± 3.6	NI
3	Horvath et al., 2017 [28]	365	–	Parkinson's disease (treatment not mentioned/probably usual care)	9.7 ± 6.4	72.3 ± 30.9 (I + II + III + IV)	Mild (HYS 1 and 2) −51.4% Moderate (HYS 3) – 25.5% Severe (HYS 4 and 5) −23.1%	–	816.7 ± 226.7		6.2 ± 4.1	On period
4	Horvath et al., 2015 [30]	260	64.4 (9.2)	Parkinson's disease (Medical, DBS)	9.2 ± 6.1	78.5 ± 26.9 (I + II + III + IV)	Mild (HYS 1 and 2) −59.6% Moderate (HYS 3) – 26.9% Severe (HYS 4 and 5) −13.5%	–	787.5 ± 579.7		–	On period
5	Sanchez-Ferro et al., 2018 (81)	61	–	Parkinson's disease (Levodopa)	–	–	–	–	Median (IQR) – 150 (100–266.9)		–	On period
6	Hauser et al., 2014 [14] Data from Hauser 2010	1056	–	Parkinson's disease (early and advanced) (Pramipexole ER and IR)	Placebo – 0.8 ± 1.1 ER group – 1.1 ± 1.3 IR group – 0.9 ± 1.2	Placebo – 30.1 ± 17.0 ER group – 30.4 ± 13.4 IR group – 28.2 ± 11.9 (II + III)	Modified HYS (1–1.5) Placebo – 28% ER group – 29.2% IR group – 26.2% Modified HYS [2,3] Placebo – 72% ER group – 70.8% IR group – 73.8%	–	–		–	On period
	From Poewe 2011 Data from Poewe 2011				Placebo – 0.9 ± 1.0 ER group – 1.0 ± 1.2 IR group – 1.2 ± 1.4	–	Modified HYS (1–1.5) Placebo – 29.1% ER group – 33.6% IR group – 29.6% Modified HYS [2,3] Placebo – 70.9% ER group – 66.4% IR group – 70.4%	–	–		–	–
7	Hauser et al., 2011 [16] Dta from the TEMPO study	876	–	Parkinson's disease (Rasagiline)	Placebo – 0.94 ± 1.10 Rasagiline 1 mg/day – 0.92 ± 1.24 Rasagiline 2 mg/day- 1.15 ± 1.32	–	Mean HYS – Placebo – 1.9 ± 0.5 Rasagiline 1 mg/day – 1.9 ± 0.5 Rasgiline 2 mg/day – 1.9 ± 0.5	–	–	–	–	–
	Dat from PRESTO study				Placebo – 9.7 ± 4.9 Rasagiline 0.5 mg/day – 9.3 ± 5.6 Rasagiline 1 mg/day- 8.8 ± 5.4	–	Mean HYS – Placebo – 2.1 ± 0.7 Rasagiline 0.5 mg/day – 2.0 ± 0.6 Rasagiline 1 mg/day – 2.0 ± 0.6	–	Placebo - 821 ± 485 Rasagiline 0.5 mg/day - 750 ± 379 Rasagiline - 815 ± 471	Daily Off time – Plcebo – 6.0 ± 2.2 Rasagiline 0.5 mg/day – 6.0 ± 2.0 Rasagiline 1 mg/day – 6.3 ± 2.6	–	On period
8	Shulman et al., 2010 [35]	653	65.9 [40]	Parkinson's disease (treatment not mentioned/probably usual care)	6.3 ± 5.8	41.0 ± 20.5	Mean HYS - 2.3 ± 0.9	NI	NI	NI	NI	NI
9	Honig et al., 2009 [38]	22	58.6 (9.1)	Parkinson's disease (Intrajejunal levodopa infusion)	15.3 ± 5.9	19.1 ± 14.0	NI	NI	NI	NI	NI	Off period
10	Steffen and seney et al., 2008 [36]	37	71	Parkinson's disease (majority received levodopa, n = 31, no mention about treatment in others)	NI	33 ± 16	Median HYS - 2 (Range 1–4)	NI	NI	NI	NI	NI
11	Martinez- et al., 2006 [33]	87	67.6 (9.8)	Parkinson's disease (Levodopa, Dopamine Agonist, Selegiline, Amantadine)	6.6 ± 4.8	UPDRS ADL - 11.6 ± 6.7	Medain HYS - 2 (1.5–2.5)	NI	NI	NI	NI	NI
12	Schrag et al., 2006 [15]	603	–	Parkinson's disease (Ropinirole, Bromocriptine, Levodopa)								
	a. Data from Rascol et al.,				Ropirinol Group. - 30 ± 34, L -Dopa group - 29 ± 27	Ropirinol Group - 21.5 ± 10.5, L-Dopa Group - 21.7 ± 11.3	HYS distribution, Ropirinol group: I - 12.8%, I.5–15.1%, II- 36.9%, Stage II.5–25.7, Stage III - 9.5%; HYS distribution, L Dopa: I - 22.8 %, I.5–9.0%, II- 37.1%, Stage II.5–21.3%, Stage III - 10.1	NI	NI	NI	NI	NI
	b. Data from Korcyzn et al.				Non-selegiline group: Ropirinol subgroup - 21 ± 21, Bromocriptine subgroup - 26 ± 24; Selegiline group: Ropirinol subgroup - 26 ± 20, Bromocriptine subgroup - 29 ± 28	Non-selegiline group: Ropirinol subgroup - 23.9 ± 9.6, Bromocriptine subgroup - 22.2 ± 10.6; Selegiline group: Ropirinol subgroup - 22.9 ± 12.6, Bromocriptine subgroup - 23.4 ± 11.0	HYS distribution,Non-selegiline group: Ropirinol subgroup: I - 9.6%, I.5–9.6%, II- 40%, Stage II.5–31.1, Stage III - 9.6%; HYS distribution,Bromocriptine subgroup: I - 14.6 %, I.5–8.2%, II- 45.5%, Stage II.5–20.9%, Stage III - 10.9; HYS distribution; Selegiline group: Ropirinol subgroup: I - 17.0%, I.5–13.2%, II- 37.3%, Stage II.5–17.0, Stage III - 15.1%; HYS distribution,Bromocriptine subgroup: I - 12.3 %, I.5–15.8%, II- 38.6%, Stage II.5–28.1%, Stage III - 5.3	NI	NI	NI	NI	NI
13	Mestre et al., 2015 [13]	11	64 [[41], [42], [43], [44], [45], [46], [47]]	Parkinson's disease (Levodopa/vehicle)	9.0 [[7], [8], [40], [48], [49], [50]]	MDS UPDRS Part III(practically defined OFF) - (24.2–34.8)	Hoehn Yahr (ON) - Stage II - 7, Stage (2.5–3) - 2; UDysRS Part 1 b - 14.0 (11.0–16.0) Time since Onset of Dyskinesia – 4.0 (range 2–6 years) Time spent with dyskinesia - 1–2 years - 7; 3–4 years - 2	NI	1232 (1025–1048)	–	–	–
14	Horvath et al., 2017 [29]	365	–	Parkinson's disease (Medical, DBS)	9.7 ± 6.4	72.3 ± 30.9	HYS distribution, Mild (HYS 1 and 2) - 187 ± 51.4; Moderate (HYS 3) - 94 ± 25.5; Severe (HYS 4 and 5) - 84 ± 23.1	NI	584.1 ± 521.3	NI	6.2 ± 4.1	NI
15	Fitzpatrick et al., 2004 [34]	946	–	Parkinson's disease	8.6 (range: 0–40)	NI	NI	NI	NI	NI	NI	NI
16	Peto et al., 2001 [37]	728	70.4	Parkinson's disease	8.6 (range: 0–40)	NI	NI	NI	NI	NI	NI	NI
17	Luo et al., 2009 [32]	96	58.4 (8.8)	Parkinson's disease	5.3 ± 4.3	UPDRS motor scores 19.5 ± 9.5	Median HYS - 2 (IQR 2.0–2.0)	NI	NI	NI	NI	NI
18	Horvath et al., 2015 [31]	413	64.83 (9.2)	Parkinson's disease (Levodopa, dopamine agoinst, benzodiazepine)	9.91 ± 5.99	77.2 ± 25.8	HYS I - 68, HYS II - 125, HYS III - 170, HYS IV - 35, HYS V - 15	NI	Levodopa - 585.4 ± 472.1, Dopamine agonist - 215 ± 244.9	NI	9.91 ± 5.99	77.2 ± 25.8
19	Godi et al., 2020 [24]	148	70.4 (8.4)	Parkinson's disease (Rehabilitation)	8.6 ± 5.4	NI	Mean HYS - 2.7 ± 0.7	NI	NI	NI	NI	NI
20	Fietzek et al., 2020 [9]	37	68.2 (8.5)	Parkinson's disease (Levodopa)	12.9 ± 6.4	NI	HYS (OFF) - 3.1 ± 0.6, Freezing of Gait (FOG) Questionnaire- 13.5 ± 3.9	NI	NI	NI	NI	NI
21	Henderson et al., 2019 [25]	–	–	Parkinson's disease	Mean years managing PD - Round 1 N - 14 ± 9.3, Round 2 N - 14 ± 9.7	NI	Delphi method used, Clinical Neurologist - Round 1 N, n (%) - 13 [51]; Clinical Geriatrician - Round 2 N, n (%) - 21 [11]	NI	NI	NI	NI	NA
22	Kluger et al., 2017 [17]	94	65.4	Parkinson's disease	24.2 ± 10.0	HYS distribution, I - 6 (6.5%), I.5–9 (9.7%), II- 28 (30.1%), Stage II.5–30 (32.3%), Stage III - 16 (17.2%), Stage IV - 4 (4.3%); MFIS total: 49.4 ± 11.7	NI	594.5 ± 434.4	NI	NI	NI	24.2 ± 10.0

Table 3.2 shows studies assessing patients with other movement disorders										
S no.	**Author, Year**	**No. of patients**	**Mean Age**	**Disease (specific treatment)**	**Disease Duration in years**	**Age at disease onset (years)**	**Disease Severity, n(%) or points (Mean** ± **SD)**	**Time from disease onset to diagnosis, years**	**Time from diagnosis to treatment, years**	**Major Treatment received**
23	Krismer et al., 2016 [11]	174	65 (8.5)	MSA-P (Rasagiline)	Placebo - 3.7 ± 2.4 Rasagiline - 4.2 ± 2.4		Total UMSARS; Placebo - 36.4 ± 9.1, Rasagiline – 38.1 ± 8.6	–	–	Rasagiline
24	Hewer et al., 2016 [12]	313	68 (6.6)	PSP (Duvanetide)			PSPRS -			Levodopa use – 131 (42%)
25	Hauser et al., 2022 [18]	256	56.8 (10.19)	Tardive Dyskinesia	5.8 ± 6.01	–	9.9 ± 3.0	–	–	
26	Barkey et al., 2021 [23]	295	–	Tardive dyskinesia (Deutetrabenazine)	–	–	–	–	–	Deutetrabenazine
27	Stacy et al., 2019 [10]	373	–	Tardive dyskinesia (Valbenazine)	–	–	AIMS total score: Placebo – 8.9 ± 4.4 Valbenazine low dose – 9.0 ± 4.2 Valbenazine high dose – 9.5 ± 3.6	–	–	Valbenazine 40–100 mg
28	Esther Cubo et al., 2021 [39]	12	44.5 (range:27–68)	patients with genetically determined dystonia (Tor1A and THAP1)	–	11.1 (range 7–27).	BFMDRS-DS – 9.95 ± 3.89 BFMDRS -MS – 39.87 ± 14.04	–	–	All 12 patients underwent bilateral GPi DBS
29	Pinter et al., 2020 [22]	198	46.1 (16.2)	Primary dystonia (Medical, DBS)	10.6 ± 10.4	–	BFMD-RS - 39.3 ± 26.2 BFMD-DS - 8.6 ± 6.6	–	–	–
30	Dashtipour et al., 2019 [21]	1046	58	Cervical dystonia (OnabotulinumtoxinA)	–	49.0 ± 16.7	Mild 345 (33.2) Moderate 548 (52.7) Severe 146 (14.1)	5.0 ± 8.1	1.1 ± 4.5	Prior treatment with botulinum toxin, n (%) - 380 (36.5)
31	Espay et al., 2018 [20]	304	NM	Cervical dystonia (Abobotulinum toxinA)	–	48.9 ± 15.6	Most patients (67.2%) had a mixed-type CD with 25.6% having had pure torticollis.	–	–	Prior treatment with botulinum toxin, n (%) - 252 (72.6)
32	Pinter et al., 2019 [19]	248	58.7 (16.7)	Essential tremor	11.8 ± 11.3	39.7 ± 24.1	FTMTRS Total score = 42.1 ± 22.7 points	–	–	–

BFMD –RS -, BFMD – DS - Burke-Fahn-Marsden Dystonia Rating Scale-Disability Score, BFMDRS-MS = Burke-Fahn-Marsden Dystonia Rating Scale-Motor Score, FTMTRS - Fahn-Tolosa-Marin Tremor Rating Scale, GPi - globus pallidum internum (GPi), DBS – Deep Brain Stimulation, PSPRS – Progressive Supranuclear Play Rating Scale.

**Table 4:** Shows the methods used, the nature of the scales and the MCID thresholds for the various scales reported by studies in the literature.

S no.	Author, Year	Disease (specific treatment)	Scale	Subscale/dimension	Generic	Specific	Anchor based	Distribution based	MCID threshold
1	Makkos et al., 2019 [1]	Parkinson's disease (Medical, DBS)	UPDRS Part IV, UDysRS	x	x	Yes	Yes	Yes	UDysRS part I: Remission of dyskinesia: 2.1 points decrease; Onset of dyskinesia 1.8 points increase; UDysRS part III: Remission of dyskinesia: 1.8 points decrease; Onset of dyskinesia 1.7 points increase; UPDRS Part IV: Improvement 0.9 points and Worsening 0.8 points
2	Makkos et al., 2018 [27]	Parkinson's disease (treatment not mentioned/probably usual care)	MDS-UPDRS scale	MDS-UPDRS – Total (I,II,III,IV), (II,III), (I,II,III)	x	Yes	Yes	x	MDS-UPDRS: Total (I + II + III + IV) Improvement 7.1, worsening 6.3; MDS-UPDRS: Part II + III: Improvement >4.9, worsening >4.2; MDS-UPDRS Part I + II + III: Improvement 6.7, Worsening 5.2
3	Horvath et al., 2017 [28]	Parkinson's disease (treatment not mentioned/probably usual care)	MDS-UPDRS scale	Part I, part II, (I,II)	x	Yes	Yes	x	MDS-UPDRS: Part I: Improvement 2.64, worsening 2.45; MDS-UPDRS: Part II: Improvement 3.05, worsening 2.51,MDS-UPDRS: Part (I + II): Improvement 5.73, worsening 4.7
4	Horvath et al., 2015 [30]	Parkinson's disease (Medical, DBS)	MDS-UPDRS scale	Part III	x	Yes	Yes	Yes	Improvement: 3.25, Worsening: 4.63
5	Sanchez-Ferro et al., 2018 (81)	Parkinson's disease (Levodopa)	UPDRS-III	x	x	Yes	Yes	Yes	Improvement: (minus)4.83; Worsening: 4.38
6	Hauser et al., 2014 [14]	Parkinson's disease (early and advanced) (Pramipexole ER and IR)	UPDRS	II, III, (II,III)	x	Yes	Yes	x	EPD, Part II: Improvement (ER and IR respectively) -(minus)1.8, and −2.0; EPD, Part III: Improvement (ER and IR respectively) -(minus) 6.2, and −6.1, EPD, Part (II + III): Improvement (ER and IR respectively) -minus 8.0, and −8.1, APD, Part II: Improvement (ER and IR respectively) -(minus)1.8, and −2.3, APD, Part III: Improvement (ER and IR respectively) -(minus) 5.2, and −6.5, APD, Part (II + III): Improvement (ER and IR respectively) -(minus)7.1, and −8.8
7	Hauser et al., 2011 [16]	Parkinson's disease (Rasagiline)	UPDRS	Total, ADL, Motor	x	Yes	Yes	x	UPDRS: Total (I + II + III): Improvement -(minus) 3.0, worsening 2.5, Part II: Improvement -(minus)0.7, Worsening 1, Part III: Improvement -(minus)2.4, Worsening 1.5
8	Shulman et al., 2010 [35]	Parkinson's disease (treatment not mentioned/probably usual care)	UPDRS	Total, motor	x	Yes	Yes	Yes	UPDRS: Motor (part II): Improvement 2.3 to 2.7, UPDRS Total (Part I + II + III): Improvement 4.3 to 4.5
9	Honig et al., 2009 [38]	Parkinson's disease (Intrajejunal levodopa infusion)	UPDRS (motor, complications, dyskinesia), PDSS, PDQ-8, NMSS	x	x	Yes	x	Yes	UPDRS Part III: Both Improvement and worsening, 0.25 SD: 3.5, 0.5 SD: 7, 10% MPS(Maximum Possible Score (MPS)): 10.3, UPDRS Part IV: Both Improvement and worsening, 0.25 SD: 1.5, 0.5 SD: 7, 10% MPS: 2.3, UPDRS Dyskinesia score (Items 32 to 35 of the UPDRS): Both Improvement and worsening, 0.25 SD: 0.6, 0.5 SD: 1.3, 10% MPS: 1.6, PDSS 6.6 points PDQ-8- Both improvement and worsening 9.2 points NMSS-total 28.2
10	Steffen and seney et al., 2008 [36]	Parkinson's disease (majority received levodopa, n = 31, no mention about treatment in others)	UPDRS,BBS, Forward and backward functional reach, RT, ABC Scale, 6MWT, comfortable and fast gait speed, TUG test, SF-36	x	x	Yes	x	Yes	UPDRS Part 1:Improvement 2, UPDRS Part II: Improvement 4, UPDRS Part III: Improvement 11, UPDRS Part (I + II + III):Improvement 13 points, BBS 5 ABC 13 Functional reach test: Forward 9, Backward 7, ABC 13 points, RT: Eyes open 10, closed 19, 6MWT 82 m, comfortable and fast gait speed −0.18 m/s, and 0.25 m/s, respectively, TUG test 11 s, SF 36 19–45%
11	Martinez- et al., 2006 [33]	Parkinson's disease (Levodopa, Dopamine Agonist, Selegiline, Amantadine)	UPDRS-ADL, SES), ISAPD	x	x	Yes	x	Yes	UPDRS-Part II(ADL)- (worsening)+2, ISAPD +1.5, SES –(minus) 6
12	Schrag et al., 2006 [15]	Parkinson's disease (Ropinirole, Bromocriptine, Levodopa)	UPDRS	Total, ADL, Motor	x	Yes	Yes	x	All HY stages - UPDRS Part (I + II + III) - Improvement – 8 points, All HY stages - UPDRS Part III – Improvement – 5 points, HY stages I/I.5 and II – UPDRS Part II – Improvement – 2 points, HY stages II·5/III – UPDRS Part II – Improvement – 3 points
13	Mestre et al., 2015 [13]	Parkinson's disease (Levodopa/vehicle)	UDysRS	Part III	x	Yes	Yes	x	UDysRS part III: Remission of dyskinesia: 2.32 decrease; Onset of dyskinesia 2.76 points increase
14	Horvath et al., 2017 [29]	Parkinson's disease (Medical, DBS)	PDQ-39, PDQ-8	x	x	Yes	Yes	Yes	PDQ-39-SI: Improvement –(minus) 4.72, worsening 4.22 PDQ-8: Improvement –(minus) 5.94, worsening 4.91
15	Fitzpatrick et al., 2004 [34]	Parkinson's disease	PDQ-39	Mobility, activities of daily living, emotional well-being, stigma, social support, cognition, communication, and bodily pain.	x	Yes	x	Yes	Total score: Improvement 1.95, worse –(minus) 2.65
16	Peto et al., 2001 [37]	Parkinson's disease	PDQ-39	x	x	Yes	Yes	x	Improvement: (minus)0.6; worsening –(minus)1.6
17	Luo et al., 2009 [32]	Parkinson's disease	PDQ-8	x	x	Yes	Yes	x	Worsening 5.8 to 7.4 points
18	Horvath et al., 2015 [31]	Parkinson's disease (Levodopa, dopamine agoinst, benzodiazepine)	PDSS-2	x	x	Yes	Yes	Yes	Improvement: 3.44, worsening 2.07
19	Godi et al., 2020 [24]	Parkinson's disease (Rehabilitation)	Mini-BESTest	x	x	Yes	Yes	Yes	4
20	Fietzek et al., 2020 [9]	Parkinson's disease (Levodopa)	FOG	x	x	Yes	Yes	x	3 points
21	Henderson et al., 2019 [25]	Parkinson's disease	Falls rate	x	Yes	x	x	x	25% (IQR 20–25%)
22	Kluger et al., 2017 [17]	Parkinson's disease	Modified Fatigue Impact Scale (MFIS).	MFIS total, MFIS cognitive, and MFIS physical	Yes	x	Yes	x	13.8, 6.8 and 6.2 for the MFIS total, MFIS cognitive, and MFIS physical, respectively.
23	Krismer et al., 2016 [11]	MSA-P (Rasagiline)	UMSARS	Total, ADL, Motor	x	Yes	Yes	x	UMSARS: Total worsening 3.5 ADL subscale worsening 1.5 Motor examination subscale worsening 1.5
24	Hewer et al., 2016 [12]	PSP (Duvanetide)	PSPRS	x	x	Yes	Yes	x	Worsening 5.7 points
25	Hauser et al., 2022 [18]	Tardive Dyskinesia	Abnormal Involuntary Movement Scale (AIMS)	x	Yes	x	Yes	x	−2 points
26	Barkey et al., 2021 [23]	Tardive dyskinesia (Deutetrabenazine)	Abnormal Involuntary Movement Scale (AIMS)	x	x	Yes	Yes	x	3
27	Stacy et al., 2019 [10]	Tardive dyskinesia (Valbenazine)	Abnormal Involuntary Movement Scale (AIMS)	x	x	Yes	Yes	Yes	2
28	Esther Cubo et al., 2021 [39]	patients with genetically determined dystonia (Tor1A and THAP1)	BFMD-RS	x	Yes	Yes	Yes	x	>77% reduction in BFMD RS for improvement
29	Pinter et al., 2020 [22]	Primary dystonia (Medical, DBS)	BFMD-RS, BFMD-DS, SF-36	x	Yes	Yes	Yes	Yes	BFMD-RS: Improvement >16.6%, worsening >21.5% BFMD-DS: Improvement or worsening >0.5 points SF-36: Improvement >7.5 points, worsening >8.5 points
30	Dashtipour et al., 2019 [21]	Cervical dystonia (OnabotulinumtoxinA)	TWSTRS	x	x	Yes	Yes	x	≥8
31	Espay et al., 2018 [20]	Cervical dystonia (Abobotulinum toxinA)	TWSTRS	x	x	Yes	Yes	x	−11.9
32	Pinter et al., 2019 [19]	Essential tremor	QUEST	x	x	Yes	Yes	Yes	Improvement >4.47; Worsening >4.98

**Table 5:** Shows the various anchors, viewpoints, statistical methods, and the type of method used to determine the respective MCID thresholds.

Sl. No.	Author, Year	Scales	Number of anchors	Anchor (s)	View point	Cutoffs used	Statistical methods	Number of distribution criteria used	Distribution criteria used	Delphi method
1	Makkos et al., 2019 [1]	UPDRS Part IV, UDysRS	2	PGI-I	Patient	Response on a scale	ROC	1	0.2 Effect size	
2	Makkos et al., 2018 [27]	MDS-UPDRS scale	1	PGI-I	Patient	No change vs minimal improvement and minimal worsening	Regression analysis	x	x	
3	Horvath et al., 2017 [28]	MDS-UPDRS scale	1	PGI-I	Patient	No change vs minimal improvement and minimal worsening	ROC	x	x	
4	Horvath et al., 2015 [30]	MDS-UPDRS scale	1	CGI-I	Patient	Response on a scale (7-point Likert scale)	Mean change approach, ROC	1	Effect size	
5	Sanchez-Ferro et al., 2018 (81)	UPDRS-III	3	Physical Health item of the SLS-6, Disability and motor signs item of the CISI-PD	Patient, Clinician	Change of atleast one point	ROC	1	SD	
6	Hauser et al., 2014 [14]	UPDRS	2	PGI-I, CGI-I	Patient, Clinician	Response on a scale (7-point Likert scale)	Mean change	x	x	
7	Hauser et al., 2011 [16]	UPDRS	1	CGI-I	Clinician	Response on a scale (7-point Likert scale)	ROC	x	x	
8	Shulman et al., 2010 [35]	UPDRS	3	Disability (SE scale), Disease stage (HY), Quality of life (SF-12)	Patient, Clinician	SF-12: 1SD, HY (1 stage), SE scale: 10% change	Mean change	1	SD	
9	Honig et al., 2009 [38]	UPDRS (motor, complications, dyskinesia), PDSS, PDQ-8, NMSS	x	x	x	x	x	1	10% of maximum possible score, 0.5 SD, and 0.25 SD	
10	Steffen and seney et al., 2008 [36]	UPDRS,BBS, Forward and backward functional reach, RT, ABC Scale, 6MWT, comfortable and fast gait speed, TUG test, SF-36	x	x	x	x	x	1	SEM	
11	Martinez-Martin et al., 2006 [33]	UPDRS-ADL, SES), ISAPD	x	x	x	x	x	2	Effect size, SRM	
12	Schrag et al., 2006 [15]	UPDRS	1	CGI-I	Clinician	Response on a scale (7-point Likert scale)	Mean change	x	x	
13	Mestre et al., 2015 [13]	UDysRS	3	Onset, maximum intensity, remission of dyskinsesia	Patient	Response on a scale (5-point Likert scale)	Median change, ROC	x	x	
14	Horvath et al., 2017 [29]	PDQ-39, PDQ-8	1	PGI-I	Patient	Response on a scale (7-point Likert scale)	ROC	1	Effect size	
15	Fitzpatrick et al., 2004 [34]	PDQ-39	x	x	x	x	x	2	SEM, SD	
16	Peto et al., 2001 [37]	PDQ-39	1	CGI-I	Clinician	No change vs minimal improvement and minimal worsening	Mean change approach, ROC	1	Effect size	
17	Luo et al., 2009 [32]	PDQ-8	1	Questionnarie	Patient	Response on a scale (5-point Likert scale)	Mean change	x	x	
18	Horvath et al., 2015 [31]	PDSS-2	1	Questionnarie	Patient	Response on a scale (5-point Likert scale)	Mean change	x	x	
19	Godi et al., 2020 [24]	Mini-BESTest	2	Global rating of change, ABC-5L	Patient, Clinician	Meaningful change (GRC>3, ABC-5L > 10%) or not	Mean change approach, ROC	1	0.5SD	
20	Fietzek et al., 2020 [9]	FOG	1	FOG questionnaire	Patient, Clinician	Response on a scale (7-point Likert scale)	ROC	x	x	
21	Henderson et al., 2019 [25]	Falls rate	x	x	x	x	x	x	x	
22	Kluger et al., 2017 [17]	Modified Fatigue Impact Scale (MFIS).	1	CGI-I	Clinican	Response on a 7-point scale	Mean change	x	x	
23	Krismer et al., 2016 [11]	UMSARS	1	CGI-I	Clinician	Response on a scale (7-point Likert scale)	ROC	x	x	
24	Hewer et al., 2016 [12]	PSPRS	1	CGIC	Clinician	Response on a scale	Mean change	x	x	
25	Hauser et al., 2022 [18]	Abnormal Involuntary Movement Scale (AIMS)	2	PGI-C, CGI-C	Patient, Clinican	Improvement or worsening	Mean change	x	x	
26	Barkey et al., 2021 [23]	Abnormal Involuntary Movement Scale (AIMS)	2	PGIC, CGIC	Patient, Clinician	Improved or not improved	Mean change	x	x	
27	Stacy et al., 2019 [10]	Abnormal Involuntary Movement Scale (AIMS)	2	PGIC; CGI-TD	Patient, Clinician	Response on a scale (7-point Likert scale)	Mean change	1	0.5 SD	
28	Esther Cubo et al., 2021 [39]	BFMD-RS	1	CGI-C	Clinician	Improvement	Mean change	x	x	
29	Pinter et al., 2020 [22]	BFMD-RS, BFMD-DS, SF-36	1	PGI-I	Patient	Response on a scale (7-point Likert scale)	Change difference, Regression analysis	1	Effect size	
30	Dashtipour et al., 2019 [21]	TWSTRS	2	PGIC, CGIC	Patient, Clinician	Response on a scale (7-point Likert scale)	Regression analysis	x	x	
31	Espay et al., 2018 [20]	TWSTRS	1	PGIC	Patient	Response on a scale (7-point Likert scale)	Regression analysis	x	x	
32	Pinter et al., 2019 [19]	QUEST	1	PGI-I	Patient	Improvement or worsening	ROC, mean change approach	1	SEM, Effect size	

Patient Global Impression of Improvement (PGI-I); Patient global impression of change (PGIC); Clinician global impression of change (CGIC); Activities-Specific Balance Confidence scale 5-levels (ABC-5L); Clinician global impression of change-Tardive dyskinesia (CGI-TD); Physical Health item of the Satisfaction with Life Scale (SLS-6), Disability item of the Clinical Impression of Severity Index (CISI-PD), Motor Signs item of the Clinical Impression of Severity Index (CISI-PD), SRM - Standardized Response Mean.

#### 2.3.1 MCID thresholds of scales reported in Parkinson's disease research

##### 2.3.1.1 MCID in Unified Parkinson's Disease Rating Scale (UPDRS) and MDS (Movement Disorder Society)-UPDRS scale

UPDRS was developed in the 1980s [48] and has since become the most widely used clinical rating scale for Parkinson's disease (PD) [49,40]. In 2001, the MDS recommended the development of a new version of the UPDRS that would include clinically relevant PD-related problems that were not well-captured in the original version, while retaining the original scale's four-part structure. Accordingly, MDS published a revision of the UPDRS in 2007, known as the MDS-UPDRS. The four sections comprised of: Parts I and II: Non-Motor and Motor Experiences of Daily Living; Part III: Motor Examination; and Part IV: Motor Complications. The MDS-UPDRS has been shown to have good validity and reliability [50] and to be more responsive than the original UPDRS [52]. The MCID thresholds of the MDS UPDS scale, which is the preferred scale being used by the majority of current studies is discussed herein, while those of the original UPDRS scale are provided in Table 3, Table 4 and Table 5 (Sl.no. 5–12), and discussed in detail in the supplementary appendix.

Makkos et al., 2019, estimated the MCID for MDS-UPDRS Part IV, in addition to the Unified Dyskinesia Rating Scale (UDysRS). The study analyzed 1044 paired investigations of 436 patients using both anchor- (PGI I and CGI I as anchors) and distribution- (0.2 effect size) based methods. A change of 0.9 points and 0.8 points were found to be clinically significant for improvement and worsening respectively for UPDRS Part IV [1].

Makkos et al., 2018 evaluated the MCID threshold values of various MDS-UPDRS-based composite scores on 452 patients of PD who underwent 1113 sequenced examinations, using anchor-based method (using PGI-I and CGI-I as anchors). The MCID thresholds for MDS-UPDRS Part II + III, I + II + III and total (I + II + III + IV) score were 4.9 points and 4.2 points, 6.7 and 5.2 points and 7.1 and 6.3 points for improvement and deterioration, respectively [27].

Horvath et al., 2017, evaluated the MCID thresholds for MDS-UPDRS Parts I and II and composite score Part I + II, in 985 paired investigations of 365 patients using three different techniques. Anchor-based method was used using PGI-I as anchor. The MCID thresholds for MDS-UPDRS Part I, Part II and Part I + II were 2.64 points and 2.45 points, 3.05 and 2.51 points, and 5.73 points and 4.70 points for improvement and deterioration, respectively [28].

Horvath et al., 2015, assessed the MCID of MDS UPDRS Part III in 728 paired investigations of 260 patients with PD. Both anchor-(CGI-I as anchor) and distribution-(effect size) based methods were used. MCID for MDS-UPDRS Part III were estimated at - (minus) 3.25 points and 4.63 points for improvement and worsening, respectively [30].

**Fig. 4:**
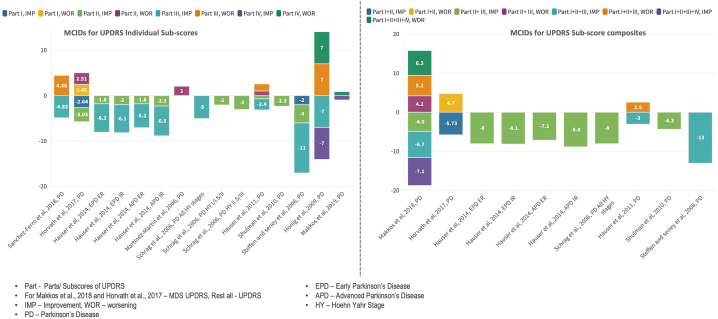
Fig. 4 shows a pictorial summary of various MCID thresholds reported for UPDRS scale reported till now in the literature. MCID thresholds for improvement are depicted below the neutral line (as they represent improvement, hence negative values) while those for worsening are depicted above the neutral line.

In summary, all the four studies reporting on MDS-UPDRS had assessed different sub-parts of MDS-UPDRS, reporting the change of 2.64, 3.05, 3.25 and 0.9 points to detect clinically meaningful improvement and 2.45,2.51,4.63 and 0.8 points to detect clinically meaningful worsening, for the Part I, II, III and IV, respectively. For the composites of Part I + II, II + III, Part I + II + III and Part I + II + III + IV, the MCID thresholds reported for clinically meaningful improvement were 5.73, 4.9, 6.7 and 7.1 respectively; while those reported for clinically meaningful worsening were 4.7, 4.2, 5.2 and 6.3, respectively (Table 3, Table 4, Table 5). A pictorial summary of the various MCID thresholds reported for the UPDRS scale is shown in Fig. 4.

##### 2.3.1.2 MCID for Unified Dyskinesia Rating Scale (UDysRS)

UDysRS is a clinical rating scale used to evaluate involuntary movements in patients with treated Parkinson's disease [53,54]. UDysRS has been found to be clinimetrically sound for dyskinesia in Parkinson's disease, demonstrating acceptable levels of internal consistency, inter- and intra-rater reliability, and construct validity [55,56]. It has also been demonstrated to be highly stable for individual patient's ON and OFF periods, making it a reliable estimate of scores [51].

Mestre et al., 2015, assessed the MCID for UDysRS in 11 Parkinson's disease patients with stable bothersome dyskinesia in a randomized, double-blinded, and placebo-controlled trial. An anchor-based approach was used to determine the MCID for UDysRS, with patient-reported onset, maximum intensity, and remission of dyskinesia as anchors. A median change of 11.1 points in UDysRS Part III Impairment was necessary to be considered MCID for patient-reported remission of dyskinesia from a practically defined-OFF state. Using a sensitivity and specificity-based approach, MCID for the onset of dyskinesia was estimated at a 2.76 points increase, and for the remission of dyskinesia was a 2.32 points decrease in the UDysRS Part III scale [13].

Makkos et al., 2019 also evaluated the MCID for UDysRS in addition to UPDRS (details described above). MCID for UDysRS Part I was estimated at 2.1 points decrease and 1.8 points increase; for the remission of dyskinesia and onset of dyskinesia, respectively. For UDysRS part III, MCID was: 1.8 points decrease; and 1.7 points increase; for the remission of dyskinesia and onset of dyskinesia, respectively [1].

#### 2.3.2 MCID thresholds of scales reported in other movement disorders

##### 2.3.2.1 Unified multiple system atrophy rating scale (UMSARS)

The Unified Multiple System Atrophy Rating Scale (UMSARS) is a multidimensional disease-specific rating scale that evaluates various aspects of MSA. The scale consists of four parts: Part I evaluates the motor function of the patient, Part II assesses activities of daily living, Part III evaluates autonomic function, and Part IV is used to assess global disability. Each part is scored separately, with higher scores indicating greater impairment [57]. It is a brief, multidimensional, valid scale, with good inter-rater reliability and internal consistency [58,59].

Krismer et al., 2016 assessed the MCID for UMSARS in 174 patients with early MSA with parkinsonian symptoms. They analyzed data from a previous double-blind, randomized controlled trial of rasagiline, using CGI an anchor, and the MCID thresholds for worsening were determined. The MCID for the total UMSARS, ADL part (part II) and motor part (part I) were estimated at 3.5 points, 1.5 points and 1.5 points, respectively, based on ROC [11].

##### 2.3.2.2 Progressive supranuclear palsy rating scale (PSPRS)

PSPRS is a disease-specific measure of severity in patients with progressive supranuclear palsy (PSP). It measures disability across 28 items in six domains: daily activities (by history), behaviour, bulbar, ocular motor, limb motor and gait/midline [60]. The maximum score is 100 with a mean annual increase (deterioration) of around 10 points in patients with PSP. The validity and reliability of PSPRS has been established in several large scale trials [61,62].

Hewer et al., 2016, estimated the MCID for PSPRS by analyzing data of 313 participants from a multicentre, randomized, double-blind, placebo-controlled trial of davunetide in PSP patients. An anchor-based method with CGI-C as anchor was used to determine the MCID for PSPRS and duration of treatment was 52 weeks. The MCID for PSPRS was estimated at 5.7 points for minimal worsening based on the change from baseline scores [12].

##### 2.3.2.3 Abnormal Involuntary Movement Scale (AIMS)

AIMS is a tool used by clinicians to evaluate the severity of dyskinesias in patients taking neuroleptic medications. It consists of 12 items that specifically assess orofacial movements, as well as movements in the extremities and trunk. In addition to these items, the scale also includes assessments of overall severity, incapacitation, patient awareness of the movements, and distress associated with them [63].

Hauser et al., 2022, estimated the MCID for AIMS score in patients with tardive dyskinesia (TD) with baseline total motor AIMS score ≥6 and treated with deutetrabenazine. The analysis included data from two pivotal, 12-week, placebo-controlled studies and used PGI-C and CGIC as anchors. MCID was assessed at – (minus) 2 points and –(minus) 2.1 based on the PGIC and CGIC, respectively. This suggests that a reduction in total motor AIMS score of approximately 2 is associated with clinically meaningful improvement in TD symptoms [18].

Barkay et al., 2021, estimated MCID for AIMS in 295 patients with tardive dyskinesia (TD) who were treated with deutetrabenazine. An anchor-based method was used with PGI-I and CGI-I as anchors. MCID for AIMS was estimated – (minus) 3 for improvement in TD symptoms [23].

Stacy et al., 2019, assessed the MCID of AIMS using valbenazine trial data. Both anchor-(CGIC-TD and PGIC as anchors) and distribution-(0.5 SD) based methods were used to determine MCID. Data from three 6-week double-blind, placebo-controlled trials were pooled, with a total of 373 participants included. The study estimated a 2-point decrease in AIMS total score as the MCID [10].

The range of MCID in the reported studies ranged from 2 to 3 points, with a median IQR of 2 (2,2.5).

##### 2.3.2.4 Burke-Fahn-Marsden Dystonia Rating Scale (BFMD-RS) and

Burke-Fahn-Marsden Dystonia Disability Scale (BFMD-DS).

BFMD-RS is a clinician-administered scale that assesses the severity of dystonia based on the observation of voluntary movements and posture, as well as specific tasks such as writing, speaking, or walking. The scale consists of a total of 100 points, with higher scores indicating greater severity of dystonia. BFMD-DS is a patient-reported outcome measure that assesses the impact of dystonia on daily activities, social life, and emotional well-being. The scale consists of 30 items, with scores ranging from 0 (no disability) to 120 (severe disability). The BFMD-DS has been shown to have good reliability, validity, and sensitivity to change in patients with dystonia. Overall, BFMD-RS and BFMD-DS are complementary tools that provide important information about different aspects of dystonia, including its severity and impact on patients' daily lives [64,64].

Esther Cubo et al., 2021, estimated the MCID for BFMD-RS motor subscale (MS) using physician-reported outcomes. The study assessed twelve participants using movement disorder specialist ratings of videotapes from patients with genetically determined dystonia (Tor1A and THAP1) who underwent GPi DBS. Anchor based method was used with CGIC as anchor. Those who had BFMDRS-MS score reduction of 77%, had clinically relevant improvement, while those with score reduction of 62% had no improvement (no information on MCID for worsening) [39].

Printer et al., 2020 estimated the MCID for BFMD scale on 898 paired examinations of 198 consecutive adult patients with idiopathic and inherited segmental and generalized isolated dystonia. Both anchor- and distribution-based methods were used to calculate the MCID thresholds. For BFMD-RS any improvement >16.6% or worsening >21.5% was estimated as the MCID. MCID for BFMD-DS were 0.5 points for both decline and improvement [22].

##### 2.3.2.5 Toronto Western spasmodic torticollis rating scale (TWSTRS)

The TWSTRS is a comprehensive scale that evaluates both physical and subjective aspects of cervical dystonia, with subscales that measure severity, disability, and pain [65]. It has been thoroughly tested and proven to be a reliable and valid assessment tool. Physicians' ratings of severity using the TWSTRS have been shown to correspond well with patients' reported improvements in disability and pain following treatment with botulinum toxin [66]. The TWSTRS total score falls within the range of 0–85, where a higher score indicates a more severe condition [67].

Dashtipour et al., 2019, assessed the MCID of TWSTRS scores using data from the Cervical Dystonia Patient Registry for Observation of OnabotulinumtoxinA Efficacy (CD PROBE) and an anchor-based method (PGIC as anchor). The study included 479 patients with cervical dystonia who completed all TWSTRS assessments. MCID for improvement was found to be ≥ 8 points [21].

Espay et al., 2018, assessed the MCID for TWSTRS in patients with cervical dystonia. The changes in TWSTRS from an observational study of abobotulinumtoxinA in the routine management of cervical dystonia were analyzed using the PGIC as an anchor. The study included 304 patients with a baseline TWSTRS-Total score of 43.4 ± 19.4. The MCID for the TWSTRS-Total score was found to be 11.9, but the threshold ranged from 3.2 to 18.0 depending on the baseline severity of the disease [20].

##### 2.3.2.6 The Quality of Life in Essential Tremor Questionnaire (QUEST)

QUEST is a 30-item scale developed specifically for patients with essential tremor to measure items impacting perceived quality of life (QOL) that generic QOL measures do not effectively capture, including activities of daily living that are affected by essential tremor [68]. The Quality of Life in Essential Tremor Questionnaire (QUEST) has excellent reliability and preliminary validity data are encouraging [69]. The validity is also supported by correlations between QUEST scores and self-rated tremor severity in various body parts [68].

Printer et al., 2019, assessed the MCID of QUEST on 895 paired examinations of 248 patients were included using both anchor-(PGI-I as anchor) and distribution-(SEM and ES) based approach. Patients were assessed at every 6 months for a total of median three visits. Any improvement greater than 4.47 or any worsening greater than 4.98 in the QUEST-SI was estimated as MCID [19].

##### 2.3.2.7 MCID of other miscellaneous scales

MCID thresholds of various other scales reported in movement disorders is provided in the supplementary appendix.

## 3 Discussion

The determination of clinically relevant changes in disease is a crucial aspect of using rating scales in clinical practice and research. This is known as the MCID, which reflects the smallest changes in an outcome measure that are meaningful to patients. The consistent use of MCID threshold values is important to reveal clinically important changes as opposed to merely statistically significant changes, and contributes thereby to a more reliable translation of clinical outcomes into clinical practice [22,70].

There is a growing awareness of correlating statistically significant results with clinical relevance in clinical trials to avoid misinterpretation of study findings and prevent exposing patients to unnecessary therapies [41]. The Movement Disorder Society Task Force on Rating Scales for Parkinson's Disease in 2003 had also highlighted the importance of identifying MCID thresholds for UPDRS, a very commonly used scale in PD, encouraging researchers to MCID thresholds for the same [42]. The US Food and Drug Administration also described the need to define minimally important differences on patient-reported outcome measures used to support the labeling claims of medical products [43].

This review article is focused on the importance of the MCID in measuring outcomes of movement disorders. It provides a comprehensive overview of the MCID values for various scales used in movement disorder research, which can help researchers determine the smallest clinically meaningful changes in outcome measures. The review is significant as it combines all the reported MCID scales for patients with movement disorders, making it a valuable resource for designing future clinical trials. By understanding the different MCID scales and values, researchers can select the most appropriate scales for their study and improve the accuracy and relevance of their findings.

In the field of movement disorders, it is crucial for clinical researchers to consider the clinical relevance of study results, rather than relying solely on statistical significance. The use of MCID scales can help bridge the gap between statistical significance and clinical relevance, by measuring the smallest clinically meaningful changes in outcome measures. When selecting a primary outcome measure, it is important to ensure that it is relevant to patients and that the method of determining the outcome meets modern clinimetric standards. Additionally, the nature of the scale used must be taken into consideration when determining the MCID [44]. If the scale is ordinal, the raw score difference may not accurately represent the clinical significance of the change. In such cases, the Rasch model can be used to transform the ordinal scale into an interval-based scale, providing a more accurate determination of the MCID [45].

Despite its conceptual simplicity, determining the MCID is a challenging task, and only a few scales have a well-defined MCID. For example-establishing a MCID for the MDS-UPDRS in movement disorder research is complicated by several factors. Firstly, PD symptoms can vary throughout the day, even without motor fluctuations, making it challenging to determine the natural variation in MDS-UPDRS scores. Secondly, the MDS-UPDRS has four subscales that measure different aspects of Parkinson's disease, making it difficult to determine a single MCID. Thirdly, the MCID values for PD may vary depending on the severity of the disease, with smaller differential values for patients with mild disease and larger values for those with more severe illness. This heterogeneity is well reflected in this review regarding the UPDRS scale. All four studies that reported on MDS-UPDRS had evaluated different sub-parts and composite of sub-parts of the scale in their respective studies. The patient population studied and the treatment they received also varied among these studies (Table 3, Table 4, Table 5). Thus, multiple factors can influence the estimation of MCID, and a range of values is likely to emerge from multiple studies, highlighting the importance of carefully considering the clinical relevance of any MCID values determined and using them appropriately in interpreting study results.

Another challenge in applying it to clinical trials is that the practical and financial constraints often dictate the sample size of a trial, rather than considering the MCID. In some cases, researchers may define the estimated treatment effect and MCID after calculating the maximum sample size that is feasible, potentially leading to biased assumptions. An external multidisciplinary committee may be set up to decide upon MCIDs for key outcome measures and encourage researchers to have trial data with an adequate sample size that is powered to detect the MCID [[14], [46], [47]].

Despite the challenges and controversies surrounding the determination of MCID, it remains a crucial concept in modern clinical trials. There is currently no consensus on the best method to use for determining MCID, so it is recommended that both anchor-based and distribution-based methods be used together. These methods should be seen as complementary to each other, rather than as separate entities, to provide a more comprehensive understanding of clinical relevance [71,72].

Furthermore, the patient's perspective on their own health is becoming increasingly important in clinical trials. Trial sponsors that understand the patient, their condition, and the burden of participation could improve recruitment, retention, and the value of data generated by clinical trials [[73], [74], [75]]. A successful patient-focused approach would mean that treatments and clinical trials would address aspects of disease that are most important to patients leading to a better understanding of the scope over which a disease can impact a patient's life. In doing so, previously neglected outcome domains, such as fatigue or sleep disturbances, can be identified and corrected [76,77].

## 4 Limitations

In this systematic review, we included articles published in English literature only, which may have resulted in the exclusion of relevant studies published in other languages. Considering the heterogeneity in the included studies, the results of this systematic review should be interpreted with caution. Very few studies used uniform outcome measures for estimating the MCID even within the same symptom severity scale, leading to difficulty in pooling data and effective analysis.

## 5 Conclusion

The review is significant in that it combines all MCID scales currently reported in patients with movement disorders, for the first time in literature, providing a comprehensive resource for future trials. This review highlights the need for standardized and validated MCID scales in movement disorders research to help clinicians and researchers interpret study results and to make informed decisions about patient care. The use of MCID scales can also help to ensure that future movement disorders trials are designed and conducted in a way that is most meaningful and relevant to patients.

## Disclosures

• Funding Sources and Conflict of Interest: “No specific funding was received for this work.” And “The authors declare that there are no conflicts of interest relevant to this work.”
• Financial Disclosures for the previous 12 months: “The authors declare that there are no additional disclosures to report.”

## Ethical compliance statement

Not applicable.

• Name of the institutional review board or ethics committee that approved the study – “Not applicable.”
• Declaration of patient consent — “Informed patient consent was not necessary for this work.”
• “We confirm that we have read the Journal's position on issues involved in ethical publication and affirm that this work is consistent with those guidelines.”

## Data availability statement

The authors confirm that all the data used in this study are included in the manuscript and in the supplementary appendix and any further information can be obtained from the corresponding author VYV, on request.

## CRediT authorship contribution statement

**Biswamohan Mishra:** Writing – review & editing, Writing – original draft, Visualization, Methodology, Conceptualization. **Pachipala Sudheer:** Writing – review & editing, Methodology, Data curation. **Roopa Rajan:** Writing – review & editing. **Ayush Agarwal:** Writing – review & editing. **M V Padma Srivastava:** Writing – review & editing. **Nilima Nilima:** Writing – review & editing, Validation, Methodology. **Venugopalan Y. Vishnu:** Writing – review & editing, Methodology, Conceptualization.

## Declaration of competing interest

The authors declare that they have no known competing financial interests or personal relationships that could have appeared to influence the work reported in this paper.
