# Supplementary material for: Bridging the gap between statistical significance and clinical relevance: A systematic review of minimum clinically important difference (MCID) thresholds of scales reported in movement disorders research
Source: Heliyon. 2024 Feb 20;10(5):e26479. doi: 10.1016/j.heliyon.2024.e26479 (PMC10909673; doi:10.1016/j.heliyon.2024.e26479)
Supplement: Multimedia component 1 [file mmc1.docx]

**MCID thresholds of various other scales reported in Movement Disorders**

1. **MCID thresholds of UPDRS scale**

Sánchez-Ferro et al.,2018 evaluated the MCID for the motor subscale of the UPDRS-III in early PD. The study involved 31 PD participants and 30 age- and gender-matched healthy controls who received five evaluations over six months. The MCID was defined using both distribution- and anchor-based methods, with anchors based on the Clinical Impression of Severity Index for Parkinson’s Disease (CISI-PD) and Satisfaction with Life Scale (SLS-6) scales. The study found that a five-point UPDRS-III change in either direction (Improvement: minus 4.83; Worsening: 4.38) represents a clinically meaningful difference and can guide prescription in everyday practice for PD patients.(1)

Hauser et al., 2014 assessed MCID for UPDRS scores in early PD (EPD) and advanced PD (APD) using an anchor-based method (using both PGI-I and CGI-I as anchors). The analysis included data from two previous double-blind, parallel-group trials of pramipexole ER and pramipexole IR as an active comparator (539 subjects in EPD study and 517 subjects in the APD trial).(2),(3) The MCID values for UPDRS II, III, and II+III were calculated, as well as the MCID for "OFF" time. In EPD, the MCIDs for UPDRS II, III, and II+III were -(minus)1.8 and -2.0, -6.2 and -6.1, and -8.0 and -8.1, respectively. In APD, the MCIDs for UPDRS II, III, and II+III were -1.8 and -2.3, -5.2 and -6.5, and -7.1 and -8.8, respectively. The MCID for "OFF" time was -1.0 and -1.3 hours for pramipexole ER and pramipexole IR, respectively. All MCIDs were for improvement only.(4)

Hauser et al., 2011 determined the MCID for two primary efficacy outcome measures in Parkinson's disease (PD): change in UPDRS scores and change in "off" time in patients with motor fluctuations. Data from two previous placebo-controlled, randomized clinical trials of rasagiline (TEMPO trial - 404 early PD patients, and PRESTO trial - 472 levodopa-treated patients with motor fluctuations, assessed 6 months apart) were analyzed using an anchor-based approach with the CGI-I to determine MCID. The MCID for total UPDRS score (I+II+III), Part II and Part III were–(minus) 3.5 points and 2.5 points, and -(minus)0.7 points and 1 points, and - -(minus)2.4, and 1.5, for improvement and worsening respectively. The MCID for reduction in "off" time was 1.0 hours.(5)

Schulman et al., 2010, estimated for the UPDRS on 653 patients with Parkinson's disease using both distribution-(SD) and anchor-based (using Disability (SE scale), Disease stage (HY), Quality of life (SF-12) as anchors) approaches. The minimal CID was estimated to be 2.3 -2.7 points for the UPDRS motor (Part III) score and 4.1 – 4.5 for the UPDRS total (I+II+III) score.(6)

Honig et al.,2009 evaluated MCID for UPDRS in addition to other scales in 22 patients of advanced PD who were switched from oral medication to continuous infusion of levodopa/carbidopa gel and followed for 6 months. Distribution-based approach was used to determine the MCID (criteria used being 10% of maximum possible score(MPS), 0.5 SD, and 0.25 SD). MCID using 0.25 SD for UPDRS Part III, IV and dyskinesia were 3.5, 1.5 and 0.6 points respectively. Using 0.5 SD MCIDs for the same scales were 7.0, 1.5 and 1.3 points respectively. Using 10% of the maximum possible score the MCIDs for the same scales were 10.3, 2.3 and 1.6 points respectively. All these MCID scores are for both improvement and worsening.(7)

Steffen and seney et al, 2008 assessed the MCID of UPDRS in people with parkinsonism. Thirty-seven community-dwelling adults with parkinsonism participated and were tested twice by the same raters, with 1 week between tests. Distribution based method was used with SEM as the distribution criteria. MCID for UPDRS part I, II, III and I+II+III are 2,4, 11 and 13 respectively.(8)

Martinez-Martin et al., 2006, evaluated the MCID for UPDRS-ADL(Part II) on 87 PD patients who were assessed during regular medical visits across a follow-up period of 2.6 ± 1.0 years. The distribution-based method was used to estimate MCID with Effect size, SRM being the distribution criteria used. Taking an increase of 0.5 in HY stage as the minimal

Change, the UPDRS-ADL (Part II) was estimated at +2 (worsening).(9)

Schrag et al., 2006 assessed the MCID for UPDRS in early PD after 6 months of treatment. Data from two independent randomized treatment trials over 6 months involving 603 patients with de novo PD were analyzed.(10),(11) An anchor-based method using ratings on a seven-point global clinical improvement was used to determine the MCID. The study found that a change of 5 points on the UPDRS Part III and 8 points for the UPDRS total score (Part I+II+III) represented a clinically meaningful improvement for all HY stages I to III. For the UPDRS Part II, an MCID of 2 points for HY stages I/I.5 and II and of 3 points for HY stage II.5/III was the most appropriate cutoff score.(12)

**3.3.1.3. Parkinson’s disease (PD) “quality of life” questionnaires (the PDQ-39 and PDQ-8)**

The 39-item Parkinson's Disease Questionnaire (PDQ-39) is a disease-specific instrument widely used to assess health-related quality of life (HRQoL) in patients with Parkinson's disease (PD).(13) Although the 39 items can be grouped into eight dimensions, their scores can be analyzed independently or combined to form the PDQ-39 Summary Index (PDQ-39-SI), a single value that reflects overall HRQoL. The highest score on the PDQ-39 scale is 100.(14)

Given that the assessment of PDQ-39 can be time-consuming, a shorter version of the questionnaire, PDQ-8, was developed.(15) PDQ-8 consists of eight representative items from the eight dimensions of the original scale, and numerous studies have shown that the PDQ-8-SI produces similar results to the PDQ-39-SI.(16),(17)

Furthermore, research has demonstrated that both PDQ-39 and PDQ-8 are more sensitive to longitudinal PD-related changes than general HRQoL instruments.(18),(19) Overall, while the PDQ-39 remains a widely used and validated instrument, the PDQ-8 may provide a more practical and efficient means of assessing HRQoL in PD patients without compromising sensitivity to change.(20)

Horvath et al.,2017 assessed the MCID for both PDQ-39 and PDQ-8 Summary Indices (PDQ-39-SI and PDQ-8-SI) in Parkinson's disease using anchor- (PGI-I as anchor) and distribution-(effect size) based techniques. A total of 985 paired investigations of 365 patients, treated with regular care, were included. Three different techniques were used simultaneously to calculate the MCID values. The MCID thresholds for PDQ-39-SI were –4.72 and +4.22 for detecting improvement and worsening, respectively. For PDQ-8-SI, these estimates were –5.94 and +4.91 points for detecting improvement and worsening, respectively.(21)

Fitzpatrick et al.,2004 assessed the MCID for PDQ 39 in 146 patient of Parkinson's disease who were either from the community or attending neurology outpatient clinics of six hospitals in the region. A distribution-based approach was used to estimate the MCID, with SEM and the standard error of the difference (Sdiff) being the distribution criteria. On the 0-100 reformatted scoring, for the total (overall) score an overall change of 1.95 and -(minus)2.65 indicating MCID for improvement and worsening, respectively.(22)

Peto et al., 2001, assessed the MCID for PDQ 39 using postal survey on 728 PD patients who were randomly selected from 13 local branches of the Parkinson's Disease Society. They were asked to complete the PDQ-39 on two occasions, 6 months apart. The MCID varied across the sub-components of PDQ 39 score. For the total PDQ 39 score, the MCID was estimated at –(minus)0.6 and –(minus)1.6 points, for improvement and worsening, respectively.(23)

Luo et al., 2009, assessed the MCID for PDQ-8 using an anchor-based approach. The study included 96 PD patients who completed the English or Chinese version of PDQ-8 twice during two different clinic visits, and rated their changes in health, PD severity, and overall impact of PD since their first visit using a 5-point response scale. The MCID for PDQ-8 was ranged from 5.8 to 7.4 points for worsening.(24)

Honig et al.,2009, estimated MCID for PDQ-8 in 22 patients of advanced PD who were switched from oral medication to continuous infusion of levodopa/carbidopa gel and followed for 6 months. Distribution-based approach was used to determine the MCID (criteria used being 10% of maximum possible score (MPS), 0.5 SD, and 0.25 SD).

Using 0.25 SD, 0.5 SD and 10% of the MPS, the MCID for PDQ-8 were estimated at 9.2, 4.6 and 10 points, respectively.(7)

1. **Mini-Balance Evaluation Systems Test (Mini-BESTest)**

The Mini-BESTest is a 14-item scale designed to assess dynamic balance in individuals, with a total score ranging from 0 to 28.(25) Recent studies have shown that the Mini-BESTest is superior in validity and sensitivity to other scales for assessing dynamic balance, including in patients with Parkinson's disease.(26),(27)

Godi et al.2020, assessed the MCID for Mini-BESTest in PD patients who underwent a balance-specific rehabilitation for four weeks. Both distribution-(SEM) and anchor-(Global Rating of Change (GRC) scores, rated by both patients and physiotherapists as anchor) based methods to estimate. The MCID estimated in this study was between 3.4 and 4.0 for improvement.(28)

1. **Freezing of Gait (FOG)**

The FOG score is a clinical instrument used to evaluate and quantify the severity of freezing of gait in patients with Parkinson's disease. It is based on a series of standardized maneuvers that are known to trigger episodes of freezing, such as turning, walking through narrow spaces, and initiating gait. The FOG score ranges from 0 to 32, with higher scores indicating more severe freezing of gait.(29),(30) The FOG score has been shown to be a reliable and valid tool for assessing freezing of gait in Parkinson's disease patients.(31)

Fietzek et al., 2020 assessed the MCID of FOG score in 37 PD patients in HY stages 2.5-4, and mean disease duration of 12.9 years (ranging from 2-29 years). A post-hoc blinded video rating by three raters was used to evaluate the FOG score. A 7-step therapy response scale (TRS) was constructed to anchor the relevant changes of freezing of gait according to a movement disorder expert and according to the patients’ view. The MCID for improvement based on expert clinician rating was estimated at three scale points.(32)

1. **Falls rate**

Henderson et al.,2019, assessed the MCID for falls rate in PD patients. A Delphi method was used to assemble a panel of experts in PD from academic and clinical medicine to reach a consensus of opinion. The panel consisted of 780 clinicians who had been caring for people with PD for an average of 14 years. MCID was estimated at a 25% (IQR 20-25%) relative reduction in falls rate.(33)

1. **Parkinson’s Disease Sleep Scale (PDSS) and Parkinson’s Disease Sleep Scale 2nd version (PDSS-2)**

Parkinson's disease sleep scale (PDSS) is a 15-item visual analogue scale that assesses the profile of nocturnal disturbances in Parkinson's disease (PD) patients. PDSS 2 is an expanded frequency-measuring version of PDSS to encompasses unmet needs such as sleep apnea, pain, akinesia and restless legs syndrome. It comprises 15 items that assess three domains, including motor symptoms at night, PD symptoms at night, and disturbed sleep, with scores ranging from 0 to 20 points for each domain. Higher scores indicate more sleep-related issues.(34) The maximum score of PDSS-2 is 60 points. The PDSS and PDSS-2 hav been validated in various languages and has demonstrated good reliability, validity, and responsiveness to changes in sleep quality.(35),(36),(37)

Horvath et al., 2015 assessed the MCID of PDSS-2 and its dimensions. They assessed 413 Parkinson's disease patients at baseline and after 9 months using both anchor (PGI-I as anchor) and distribution-(effect size) based methods to arrive at the MCID. MCID was estimated at a change greater than–(minus)3.44 points and greater than 2.07 points for improvement and worsening, respectively.(38)

Honig et al.,2009, estimated MCID for PDSS in addition to UDPRS as described above. Using 0.25 SD, 0.5 SD and 10% of the MPS, the MCID for PDQ-8 were estimated at 6.6 points, 3.3 points and 15.0 points, respectively.(7)

1. **Intermediate Scale for Assessment of PD (ISAPD) scale and**

**Schwab and England Scale (SES)**

The ISAPD is a rating scale used to assess the severity Parkinson's disease patients. The scale is composed of a 13-item section that assesses functional aspects and a 4-item section that assesses complications of therapy. The scale has been shown to be valid, acceptable and consistent.(39)

The SES is a measure of daily function for PD patients. The SES estimates the percentage of independence in performing activities of daily living (ADL), where 100% represents a completely independent individual and 0% represents an individual who is no longer functioning due to a life-threatening illness and loss.(40),(41),(42) SES has been found to be a reliable and valid measure of daily function for PD patients.(43),(44)

Martinez-Martin et al., 2006, assessed the MCID of SES and ISAPD in addition to UPDRS (as described above). Taking an increase of 0.5 in HY stage as the minimal change, the MCID for SES, and ISAPD are –(minus)6 and +1.5 points, respectively for worsening.(9)

1. **Berg Balance Scale (BBS), Functional reach test (FRT)- Forward and backward, Activities-specific Balance Confidence (ABC) Scale , Sharpened Romberg Test (SRT), 6-minute Walk Test (6MWT), Comfortable and fast gait speed**

**Timed Up and Go (TUG) test.**

Berg Balance Scale (BBS) test, which is a 14-item test used to measure standing balance in patients. The BBS uses ordinal scoring from 0 to 4 for each item, and higher scores indicate better balance. The test has moderate to high internal consistency and test-retest reliability.(45),(46),(47)

FRT is a quick, reliable and valid measure way to measure balance and stability in older adults and PD patients. It measures how far forward a person can reach while standing in a fixed position without losing balance. The test is performed by having the person stand next to a wall with their arm extended and then reaching forward as far as possible without taking a step.(48),(49)

ABC Scale is a questionnaire designed to measure balance confidence in specific situations. The scale consists of 16 items with scores ranging from 0% to 100%. ABC scale has been shown to have good reliability and validity in a variety of populations, including older adults, individuals with Parkinson’s disease, stroke survivors, and individuals with multiple sclerosis.(50),(51)

The Romberg Test (RT) and Sharpened Romberg Test (SRT) are tests of static balance that measure the ability to maintain balance or equilibrium with a narrowed base of support.(52),(53) While there is some research on the test-retest reliability of these tests, there are no studies that have specifically examined their reliability for subjects with Parkinson's Disease (PD), though its reliability has been found to be good in other populations like middle-aged women.(49)

The 6 Minute Walk Test (6MWT) is a submaximal exercise test used to assess both endurance and aerobic capacity in various populations including Parkinson's disease (PD) patients. The 6MWT has validity and reliability in evaluating physical function and exercise capacity in PD patients.(54),(55)

Gait speed is a valid measurement to predict community walking in Parkinson’s disease. Gait speed measures walking performance, but it doesn't account for endurance. To assess a patient's ability to adjust their walking speed, both fast and comfortable gait speeds are commonly measured.(56) Measurements of gait speed and step frequency during the 10MWT are reliable between sessions in individuals with Parkinson’s disease.(57)

The Timed Up and Go (TUG) test has been widely accepted as a standard assessment for measuring the basic functional mobility of patients with Parkinson’s disease.(58) Several basic mobility sub-tasks “Sit,” “Sit-to-Stand,” “Walk,” “Turn,” “Walk-Back,” and “Sit-Back” are included in a TUG test.(59) TUG test is a valid and reliable test for measuring mobility in PD patients.(60), (61)

Steffen and seney et al, 2008 assessed the MCID of all these scales in their study in addition to UPDRS and SF-36 (as described above). The MCID for BBS, TUG, ABC, 6MWT, comfortable gait speed and fast gait speed were estimated at 5 points, 11 seconds, 13%, 82 metres, 0.18 m/s, and 0.25 m/s, respectively. The MCID for Functional reach test (FRT): for Forward and backward were 9 cm and 7 cm, respectively. MCID for RT with eyes open and closed were 10 seconds and 19 seconds, respectively; and for SRT with eyes open and closed were 39 seconds and 19 seconds, respectively.(8)

1. **Short Form (SF 36)**

The SF-36 is a generic health-related quality of life (HRQOL) measure, also used in PD patients. In general, the SF-36 produces results of moderate to high validity regarding content, construct and criterion validity -also compared to other generic health instruments. The SF-36 also produces results of reliability and sensitivity, and finally, of responsiveness.(62),(63)

Printer et al,2020, estimated the MCID of SF36 in addition to BFMD (described above). The cut-off scores for the Physical Component Summary, the Mental Component Summary, and the Global Score of the SF-36 were 5.5 and 5.5, 6.5 and 7.5, and 7.5 and 8.5 points for clinically meaningful improvement and deterioration, respectively.(64)

In their study, Steffen and seney et al, 2008, MCID for SF 36 was estimated to vary between 19% to 45%.(8)

1. **Non-Motor Symptoms Scale NMSS**

The NMSS is a 30-item rater-based scale to assess a wide range of non-motor symptoms in patients with Parkinson’s disease (PD). The NMSS measures the severity and frequency of non-motor symptoms across nine dimensions. The scale can be used for patients at all stages of PD.(65),(66),(67) NMSS has been extensively validated internationally with good psychometric attributes and shown to be capable of detecting longitudinal changes in non-motor symptoms.(68)

Honig et al.,2009, estimated MCID for NMSS in addition to UDPRS and PDSS as described above. Using 0.25 SD, 0.5 SD and 10% of the MPS, the MCID for NMSS were estimated at 28.2, 14.1 and 36.0 points, respectively.(7)

1. **Modified Fatigue Impact Scale (MFIS).**

The Modified Fatigue Impact Scale (MFIS) is a valid multidimensional measure of fatigue in PD patients. It is a 21-item self-report measure of fatigue derived from the 40-item Fatigue Impact Scale. The MFIS assesses the effects of fatigue on physical, cognitive, and psychosocial functioning. Each item is rated based on frequency of symptoms from 0 (“Never”) to 4 (“Almost Always”) with a total score of 84, 0 to 36 for the physical subcomponent, and 0 to 40 for the cognitive subcomponent.(69),(70),(71)

Kluger et al.,2017, assessed the MCID for MFIS by performing a secondary data analysis of 94 PD participants in an acupuncture trial for PD fatigue and using an anchor-based method(CGI-C as anchor). The MCIDs values were found to be 13.8, 6.8, and 6.2 points for the MFIS total, MFIS cognitive, and MFIS physical subscores, respectively.(72)

**References**

1. Sánchez-Ferro Á, Matarazzo M, Martínez-Martín P, Martínez-Ávila JC, Gómez de la Cámara A, Giancardo L, et al. Minimal Clinically Important Difference for UPDRS-III in Daily Practice. Mov Disord Clin Pract. 2018 Aug;5(4):448–50.

2. Hauser RA, Schapira AHV, Rascol O, Barone P, Mizuno Y, Salin L, et al. Randomized, double-blind, multicenter evaluation of pramipexole extended release once daily in early Parkinson’s disease. Mov Disord Off J Mov Disord Soc. 2010 Nov 15;25(15):2542–9.

3. Schapira AHV, Barone P, Hauser RA, Mizuno Y, Rascol O, Busse M, et al. Extended-release pramipexole in advanced Parkinson disease: A randomized controlled trial. Neurology. 2011 Aug 23;77(8):767–74.

4. Hauser RA, Gordon MF, Mizuno Y, Poewe W, Barone P, Schapira AH, et al. Minimal clinically important difference in Parkinson’s disease as assessed in pivotal trials of pramipexole extended release. Park Dis. 2014;2014:467131.

5. Hauser RA, Auinger P, Parkinson Study Group. Determination of minimal clinically important change in early and advanced Parkinson’s disease. Mov Disord Off J Mov Disord Soc. 2011 Apr;26(5):813–8.

6. Shulman LM, Gruber-Baldini AL, Anderson KE, Fishman PS, Reich SG, Weiner WJ. The clinically important difference on the unified Parkinson’s disease rating scale. Arch Neurol. 2010 Jan;67(1):64–70.

7. Honig H, Antonini A, Martinez-Martin P, Forgacs I, Faye GC, Fox T, et al. Intrajejunal levodopa infusion in Parkinson’s disease: a pilot multicenter study of effects on nonmotor symptoms and quality of life. Mov Disord Off J Mov Disord Soc. 2009 Jul 30;24(10):1468–74.

8. Steffen T, Seney M. Test-retest reliability and minimal detectable change on balance and ambulation tests, the 36-item short-form health survey, and the unified Parkinson disease rating scale in people with parkinsonism. Phys Ther. 2008 Jun;88(6):733–46.

9. Martinez-Martin P, Prieto L, Forjaz MJ. Longitudinal metric properties of disability rating scales for Parkinson’s disease. Value Health J Int Soc Pharmacoeconomics Outcomes Res. 2006 Dec;9(6):386–93.

10. Korczyn AD, Brooks DJ, Brunt ER, Poewe WH, Rascol O, Stocchi F. Ropinirole versus bromocriptine in the treatment of early Parkinson’s disease: a 6-month interim report of a 3-year study. 053 Study Group. Mov Disord Off J Mov Disord Soc. 1998 Jan;13(1):46–51.

11. Rascol O, Brooks DJ, Brunt ER, Korczyn AD, Poewe WH, Stocchi F. Ropinirole in the treatment of early Parkinson’s disease: a 6-month interim report of a 5-year levodopa-controlled study. 056 Study Group. Mov Disord Off J Mov Disord Soc. 1998 Jan;13(1):39–45.

12. Schrag A, Sampaio C, Counsell N, Poewe W. Minimal clinically important change on the unified Parkinson’s disease rating scale. Mov Disord Off J Mov Disord Soc. 2006 Aug;21(8):1200–7.

13. Jenkinson C, Fitzpatrick R, Peto V, Greenhall R, Hyman N. The Parkinson’s Disease Questionnaire (PDQ-39): development and validation of a Parkinson’s disease summary index score. Age Ageing. 1997 Sep;26(5):353–7.

14. Hagell P, Nygren C. The 39 item Parkinson’s disease questionnaire (PDQ-39) revisited: implications for evidence based medicine. J Neurol Neurosurg Psychiatry. 2007 Nov 1;78(11):1191–8.

15. Jenkinson C, Fitzpatrick R, Peto V, Greenhall R, Hyman N. The PDQ-8: Development and validation of a short-form parkinson’s disease questionnaire. Psychol Health. 1997 Dec 1;12(6):805–14.

16. Katsarou Z, Bostantjopoulou S, Peto V, Kafantari A, Apostolidou E, Peitsidou E. Assessing quality of life in Parkinson’s disease: can a short-form questionnaire be useful? Mov Disord Off J Mov Disord Soc. 2004 Mar;19(3):308–12.

17. Jenkinson C, Fitzpatrick R. Cross-cultural evaluation of the short form 8-item Parkinson’s Disease Questionnaire (PDQ-8): results from America, Canada, Japan, Italy and Spain. Parkinsonism Relat Disord. 2007 Feb;13(1):22–8.

18. Reuther M, Spottke EA, Klotsche J, Riedel O, Peter H, Berger K, et al. Assessing health-related quality of life in patients with Parkinson’s disease in a prospective longitudinal study. Parkinsonism Relat Disord. 2007 Mar;13(2):108–14.

19. Luo N, Ng WY, Lau PN, Au WL, Tan LC. Responsiveness of the EQ-5D and 8-item Parkinson’s Disease Questionnaire (PDQ-8) in a 4-year follow-up study. Qual Life Res Int J Qual Life Asp Treat Care Rehabil. 2010 May;19(4):565–9.

20. Jenkinson C, Clarke C, Gray R, Hewitson P, Ives N, Morley D, et al. Comparing results from long and short form versions of the Parkinson’s disease questionnaire in a longitudinal study. Parkinsonism Relat Disord. 2015 Nov;21(11):1312–6.

21. Horváth K, Aschermann Z, Kovács M, Makkos A, Harmat M, Janszky J, et al. Changes in Quality of Life in Parkinson’s Disease: How Large Must They Be to Be Relevant? Neuroepidemiology. 2017;48(1–2):1–8.

22. Fitzpatrick R, Norquist JM, Jenkinson C. Distribution-based criteria for change in health-related quality of life in Parkinson’s disease. J Clin Epidemiol. 2004 Jan;57(1):40–4.

23. Peto V, Jenkinson C, Fitzpatrick R. Determining minimally important differences for the PDQ-39 Parkinson’s disease questionnaire. Age Ageing. 2001 Jul;30(4):299–302.

24. Luo N, Tan LCS, Zhao Y, Lau PN, Au WL, Li SC. Determination of the longitudinal validity and minimally important difference of the 8-item Parkinson’s Disease Questionnaire (PDQ-8). Mov Disord Off J Mov Disord Soc. 2009 Jan 30;24(2):183–7.

25. Using psychometric techniques to improve the Balance Evaluation Systems Test: the mini-BESTest [Internet]. [cited 2023 Apr 12]. Available from: http://medicaljournals.se/jrm/content/abstract/10.2340/16501977-0537

26. Godi M, Franchignoni F, Caligari M, Giordano A, Turcato AM, Nardone A. Comparison of Reliability, Validity, and Responsiveness of the Mini-BESTest and Berg Balance Scale in Patients With Balance Disorders. Phys Ther. 2013 Feb 1;93(2):158–67.

27. King LA, Priest KC, Salarian A, Pierce D, Horak FB. Comparing the Mini-BESTest with the Berg Balance Scale to Evaluate Balance Disorders in Parkinson’s Disease. Park Dis. 2011 Oct 24;2012:e375419.

28. Godi M, Arcolin I, Giardini M, Corna S, Schieppati M. Responsiveness and minimal clinically important difference of the Mini-BESTest in patients with Parkinson’s disease. Gait Posture. 2020 Jul;80:14–9.

29. Ziegler K, Schroeteler F, Ceballos-Baumann AO, Fietzek UM. A new rating instrument to assess festination and freezing gait in Parkinsonian patients. Mov Disord Off J Mov Disord Soc. 2010 Jun 15;25(8):1012–8.

30. Fietzek UM, Zwosta J, Schroeteler FE, Ziegler K, Ceballos-Baumann AO. Levodopa changes the severity of freezing in Parkinson’s disease. Parkinsonism Relat Disord. 2013 Oct;19(10):894–6.

31. Nieuwboer A, Rochester L, Herman T, Vandenberghe W, Emil GE, Thomaes T, et al. Reliability of the new freezing of gait questionnaire: agreement between patients with Parkinson’s disease and their carers. Gait Posture. 2009 Nov;30(4):459–63.

32. Fietzek UM, Schulz SJ, Ziegler K, Ceballos-Baumann AO. The Minimal Clinically Relevant Change of the FOG Score. J Park Dis. 2020;10(1):325–32.

33. Henderson EJ, Morgan GS, Amin J, Gaunt DM, Ben-Shlomo Y. The minimum clinically important difference (MCID) for a falls intervention in Parkinson’s: A delphi study. Parkinsonism Relat Disord. 2019 Apr;61:106–10.

34. Trenkwalder C, Kohnen R, Högl B, Metta V, Sixel-Döring F, Frauscher B, et al. Parkinson’s disease sleep scale--validation of the revised version PDSS-2. Mov Disord Off J Mov Disord Soc. 2011 Mar;26(4):644–52.

35. Martinez-Martin P, Wetmore JB, Rodríguez-Blázquez C, Arakaki T, Bernal O, Campos-Arillo V, et al. The Parkinson’s Disease Sleep Scale–2 (PDSS-2): Validation of the Spanish Version and Its Relationship With a Roommate-Based Version. Mov Disord Clin Pract. 2019;6(4):294–301.

36. Horvath K, Aschermann Z, Acs P, Bosnyak E, Deli G, Pal E, et al. Is the MDS-UPDRS a good screening tool for detecting sleep problems and daytime sleepiness in Parkinson’s disease? Park Dis [Internet]. 2014 Jan 1 [cited 2023 Apr 13]; Available from: https://go.gale.com/ps/i.do?p=HRCA&sw=w&issn=20908083&v=2.1&it=r&id=GALE%7CA427555931&sid=googleScholar&linkaccess=abs

37. Horváth K, Aschermann Z, Acs P, Deli G, Janszky J, Karádi K, et al. Test-retest validity of Parkinson’s disease sleep scale 2nd version (PDSS-2). J Park Dis. 2014;4(4):687–91.

38. Horváth K, Aschermann Z, Ács P, Deli G, Janszky J, Komoly S, et al. Minimal Clinically Important Difference on Parkinson’s Disease Sleep Scale 2nd Version. Park Dis. 2015;2015:970534.

39. Martínez-Martin P, Gil-Nagel A, Morlán Gracia L, Balseiro Gómez J, Martínez-Sarriés FJ, Bermejo F, et al. Intermediate scale for assessment of Parkinson’s disease. Characteristics and structure. Parkinsonism Relat Disord. 1995 Oct 1;1(2):97–102.

40. Schwab and England ADL scale. In: Wikipedia [Internet]. 2022 [cited 2023 Apr 13]. Available from: https://en.wikipedia.org/w/index.php?title=Schwab_and_England_ADL_scale&oldid=1120533970

41. Dementech. Your Guide to Parkinson’s Disease Disability Scales | Dementech [Internet]. Dementech Neurosciences. 2022 [cited 2023 Apr 13]. Available from: https://dementech.com/2022/11/25/your-guide-to-parkinsons-disease-disability-scales/

42. Siderowf A. Schwab and England Activities of Daily Living Scale. In: Kompoliti K, Metman LV, editors. Encyclopedia of Movement Disorders [Internet]. Oxford: Academic Press; 2010 [cited 2023 Apr 13]. p. 99–100. Available from: https://www.sciencedirect.com/science/article/pii/B9780123741059000708

43. Basit A, Noohu M, Khan F, Chevidikunnan MF. Reliability and validity of floor transfer test in subjects with idiopathic Parkinson’s disease. In 2020 [cited 2023 Apr 13]. Available from: https://www.semanticscholar.org/paper/Reliability-and-validity-of-floor-transfer-test-in-Basit-Noohu/4618324c3d0b1f5c033f1fc9e162734e76ede631

44. Fereshtehnejad SM, Farhadi F, Hadizadeh H, Shahidi GA, Delbari A, Lökk J. Cross-Cultural Validity, Reliability, and Psychometric Properties of the Persian Version of the Scales for Outcomes in Parkinson’s Disease-Psychosocial Questionnaire. Neurol Res Int. 2014 Apr 7;2014:e260684.

45. Berg K, Wood-Dauphinee S, Williams JI. The Balance Scale: reliability assessment with elderly residents and patients with an acute stroke. Scand J Rehabil Med. 1995 Mar;27(1):27–36.

46. Halsaa KE, Brovold T, Graver V, Sandvik L, Bergland A. Assessments of interrater reliability and internal consistency of the Norwegian version of the Berg Balance Scale. Arch Phys Med Rehabil. 2007 Jan;88(1):94–8.

47. Berg K, Wood-Dauphine S, Williams J i., Gayton D. Measuring balance in the elderly: preliminary development of an instrument. Physiother Can. 1989 Nov;41(6):304–11.

48. Duncan PW, Weiner DK, Chandler J, Studenski S. Functional reach: a new clinical measure of balance. J Gerontol. 1990 Nov;45(6):M192-197.

49. Franchignoni F, Tesio L, Martino MT, Ricupero C. Reliability of four simple, quantitative tests of balance and mobility in healthy elderly females. Aging Clin Exp Res. 1998 Feb 1;10(1):26–31.

50. Powell LE, Myers AM. The Activities-specific Balance Confidence (ABC) Scale. J Gerontol A Biol Sci Med Sci. 1995 Jan;50A(1):M28-34.

51. Miller WC, Deathe AB, Speechley M. Psychometric properties of the Activities-specific Balance Confidence Scale among individuals with a lower-limb amputation. Arch Phys Med Rehabil. 2003 May;84(5):656–61.

52. Johnson BG, Wright AD, Beazley MF, Harvey TC, Hillenbrand P, Imray CHE, et al. The sharpened Romberg test for assessing ataxia in mild acute mountain sickness. Wilderness Environ Med. 2005;16(2):62–6.

53. Black FO, Wall C, Rockette HE, Kitch R. Normal subject postural sway during the Romberg test. Am J Otolaryngol. 1982;3(5):309–18.

54. Kobayashi E, Himuro N, Takahashi M. Clinical utility of the 6-min walk test for patients with moderate Parkinson’s disease. Int J Rehabil Res Int Z Rehabil Rev Int Rech Readaptation. 2017 Mar;40(1):66–70.

55. Üğüt BO, Kalkan AC, Kahraman T, Dönmez Çolakoğlu B, Çakmur R, Genç A. Determinants of 6-minute walk test in people with Parkinson’s disease. Ir J Med Sci. 2023 Feb;192(1):359–67.

56. Atrsaei A, Corrà MF, Dadashi F, Vila-Chã N, Maia L, Mariani B, et al. Gait speed in clinical and daily living assessments in Parkinson’s disease patients: performance versus capacity. NPJ Park Dis. 2021 Mar 5;7(1):24.

57. Zanardi APJ, da Silva ES, Costa RR, Passos-Monteiro E, dos Santos IO, Kruel LFM, et al. Gait parameters of Parkinson’s disease compared with healthy controls: a systematic review and meta-analysis. Sci Rep. 2021 Jan 12;11(1):752.

58. Li T, Chen J, Hu C, Ma Y, Wu Z, Wan W, et al. Automatic Timed Up-and-Go Sub-Task Segmentation for Parkinson’s Disease Patients Using Video-Based Activity Classification. IEEE Trans Neural Syst Rehabil Eng Publ IEEE Eng Med Biol Soc. 2018 Nov;26(11):2189–99.

59. Zampieri C, Salarian A, Carlson-Kuhta P, Aminian K, Nutt JG, Horak FB. The instrumented timed up and go test: potential outcome measure for disease modifying therapies in Parkinson’s disease. J Neurol Neurosurg Psychiatry. 2010 Feb;81(2):171–6.

60. Greene BR, Caulfield B, Lamichhane D, Bond W, Svendsen J, Zurski C, et al. Longitudinal assessment of falls in patients with Parkinson’s disease using inertial sensors and the Timed Up and Go test. J Rehabil Assist Technol Eng. 2018 Jan 1;5:2055668317750811.

61. da Silva BA, Faria CDCM, Santos MP, Swarowsky A. Assessing Timed Up and Go in Parkinson’s disease: Reliability and validity of Timed Up and Go Assessment of biomechanical strategies. J Rehabil Med. 2017 Nov 21;49(9):723–31.

62. Hagell P, Törnqvist AL, Hobart J. Testing the SF-36 in Parkinson’s disease. Implications for reporting rating scale data. J Neurol. 2008 Feb;255(2):246–54.

63. Brown CA, Cheng EM, Hays RD, Vassar SD, Vickrey BG. SF-36 includes less Parkinson Disease (PD)-targeted content but is more responsive to change than two PD-targeted health-related quality of life measures. Qual Life Res. 2009 Nov 1;18(9):1219–37.

64. Pintér D, Janszky J, Kovács N. Minimal Clinically Important Differences for Burke-Fahn-Marsden Dystonia Rating Scale and 36-Item Short-Form Health Survey. Mov Disord Off J Mov Disord Soc. 2020 Jul;35(7):1218–23.

65. NMSS.pdf.

66. Storch A, Schneider CB, Klingelhöfer L, Odin P, Fuchs G, Jost WH, et al. Quantitative assessment of non-motor fluctuations in Parkinson’s disease using the Non-Motor Symptoms Scale (NMSS). J Neural Transm. 2015 Dec 1;122(12):1673–84.

67. Martinez–Martin P, Rodriguez-Blazquez C, Forjaz MJ. Rating Scales in Movement Disorders☆. In: Reference Module in Neuroscience and Biobehavioral Psychology [Internet]. Elsevier; 2017 [cited 2023 Apr 15]. Available from: https://www.sciencedirect.com/science/article/pii/B9780128093245007690

68. van Wamelen DJ, Martinez-Martin P, Weintraub D, Schrag A, Antonini A, Falup-Pecurariu C, et al. The Non-Motor Symptoms Scale in Parkinson’s disease: Validation and use. Acta Neurol Scand. 2021;143(1):3–12.

69. Schiehser DM, Ayers CR, Liu L, Lessig S, Song DS, Filoteo JV. Validation of the Modified Fatigue Impact Scale in Parkinson’s disease. Parkinsonism Relat Disord. 2013 Mar 1;19(3):335–8.

70. Franssen M, Winward C, Collett J, Wade D, Dawes H. Interventions for fatigue in Parkinson’s disease: A systematic review and meta-analysis. Mov Disord. 2014;29(13):1675–8.

71. Friedman JH, Alves G, Hagell P, Marinus J, Marsh L, Martinez-Martin P, et al. Fatigue rating scales critique and recommendations by the Movement Disorders Society task force on rating scales for Parkinson’s disease. Mov Disord. 2010;25(7):805–22.

72. Kluger BM, Garimella S, Garvan C. Minimal clinically important difference of the Modified Fatigue Impact Scale in Parkinson’s disease. Parkinsonism Relat Disord. 2017 Oct;43:101–4.
